# *Sucla2* Knock‐Out in Skeletal Muscle Yields Mouse Model of Mitochondrial Myopathy With Muscle Type–Specific Phenotypes

**DOI:** 10.1002/jcsm.13617

**Published:** 2024-10-31

**Authors:** Makayla S. Lancaster, Paul Hafen, Andrew S. Law, Catalina Matias, Timothy Meyer, Kathryn Fischer, Marcus Miller, Chunhai Hao, Patrick Gillespie, David McKinzie, Jeffrey J. Brault, Brett H. Graham

**Affiliations:** ^1^ Department of Medical & Molecular Genetics Indiana University School of Medicine Indianapolis Indiana USA; ^2^ Indiana Center for Musculoskeletal Health, Department of Anatomy, Cell Biology, & Physiology Indiana University School of Medicine Indianapolis Indiana USA; ^3^ Division of Science Indiana University Columbus Columbus Indiana USA; ^4^ Behavioral Phenotyping Core Indiana University School of Medicine Indianapolis Indiana USA; ^5^ Department of Pathology and Laboratory Medicine Indiana University School of Medicine Indianapolis Indiana USA

**Keywords:** contractility, extensor digitorum longus, fibre‐type switching, mitochondrial myopathy, soleus, succinyl‐CoA synthetase

## Abstract

**Background:**

Pathogenic variants in subunits of succinyl‐CoA synthetase (SCS) are associated with mitochondrial encephalomyopathy in humans. SCS catalyses the conversion of succinyl‐CoA to succinate coupled with substrate‐level phosphorylation of either ADP or GDP in the TCA cycle. This report presents a muscle‐specific conditional knock‐out (KO) mouse model of *Sucla2*, the ADP‐specific beta subunit of SCS, generating a novel in vivo model of mitochondrial myopathy.

**Methods:**

The mouse model was generated using the Cre‐Lox system, with the human skeletal actin (HSA) promoter driving Cre‐recombination of a CRISPR‐Cas9–generated *Sucla2* floxed allele within skeletal muscle. Inactivation of *Sucla2* was validated using RT‐qPCR and western blot, and both enzyme activity and serum metabolites were quantified by mass spectrometry. To characterize the model in vivo, whole‐body phenotyping was conducted, with mice undergoing a panel of strength and locomotor behavioural assays. Additionally, ex vivo contractility experiments were performed on the soleus (SOL) and extensor digitorum longus (EDL) muscles. SOL and EDL cryosections were also subject to imaging analyses to assess muscle fibre‐specific phenotypes.

**Results:**

Molecular validation confirmed 68% reduction of *Sucla2* transcript within the mutant skeletal muscle (*p* < 0.001) and 95% functionally reduced SUCLA2 protein (*p* < 0.0001). By 3 weeks of age, *Sucla2* KO mice were 44% the size of controls by body weight (*p* < 0.0001). Mutant mice also exhibited 34%–40% reduced grip strength (*p* < 0.01) and reduced spontaneous exercise, spending about 88% less cumulative time on a running wheel (*p* < 0.0001). Contractile function was also perturbed in a muscle‐specific manner; although no genotype‐specific deficiencies were seen in EDL function, SUCLA2‐deficient SOL muscles generated 40% less specific tetanic force (*p* < 0.0001), alongside slower contraction and relaxation rates (*p* < 0.001). Similarly, a SOL‐specific threefold increase in mitochondria (*p* < 0.0001) was observed, with qualitatively increased staining for both COX and SDH, and the proportion of Type 1 myosin heavy chain expressing fibres within the SOL was nearly doubled (95% increase, *p* < 0.0001) in the *Sucla2* KO mice compared with that in controls.

**Conclusions:**

SUCLA2 loss within murine skeletal muscle yields a model of SCS‐deficient mitochondrial myopathy with reduced body weight, muscle weakness and exercise intolerance. Physiological and morphological analyses of hindlimb muscles showed remarkable differences in ex vivo function and cellular consequences between the EDL and SOL muscles, with SOL muscles significantly more impacted by *Sucla2* inactivation. This novel model will provide an invaluable tool for investigations of muscle‐specific and fibre type–specific pathogenic mechanisms to better understand SCS‐deficient myopathy.

## 1 Introduction

Mitochondrial disorders are one of the most common causes of multisystem diseases, with approximately 1 in 4300 adults affected and no curative treatment available [1, 2]. Due in part to high energy demand, neurons and skeletal muscle are often affected in primary mitochondrial disease, presenting as mitochondrial encephalomyopathy, with an average onset prior to 20 years of age. Clinical symptoms include intellectual disability, basal ganglia lesions, dystonia, hypotonia, distal muscle weakness, exercise intolerance, ophthalmoplegia and occasionally respiratory distress [3, 4]. Historically, primary mitochondrial myopathies have been defined by defects in mitochondrial oxidative phosphorylation that primarily, but not exclusively, affect skeletal muscle [3]. These disorders are genetically heterogenous and can be caused by genetic variants in the mitochondrial DNA or, more frequently, by variants within the nuclear‐encoded mitochondrial proteome [5]. Succinyl‐CoA synthetase (SCS) is one such nuclear‐encoded mitochondrial enzyme with associations to mitochondrial encephalomyopathy in humans.

SCS is a tricarboxylic acid (TCA) cycle enzyme that catalyses the reversible conversion of succinyl‐CoA to succinate coupled with substrate‐level phosphorylation of either ADP or GDP. In eukaryotic organisms, SCS is a heterodimer with a single catalytic alpha‐subunit, SUCLG1, present in all tissues, and two versions of the beta‐subunit that are differentially expressed across tissues. Although GTP‐generating beta‐subunit SUCLG2 is more abundant in anabolic tissues, such as the liver and kidney, ATP‐forming SUCLA2 is the predominant beta‐isoform in tissues with high energy demand, including skeletal muscle [6]. Biallelic pathogenic variants in either *SUCLG1* or *SUCLA2* have been associated with infantile mitochondrial encephalomyopathy in humans [7, 8, 9, 10, 11]. However, the pathogenic mechanisms of SCS‐deficient mitochondrial myopathy remain poorly elucidated [3].

To date, there have been no reported laboratory models that allow for in vivo study of SCS‐deficient pathogenesis in the postnatal skeletal muscle. Rather, constitutive knock‐out (KO) of SCS subunits is embryonic lethal in published research models [12, 13, 14]. Here, by restricting *Sucla2* mutagenesis to the murine skeletal muscle via the Cre/loxP system [15, 16, 17], the development of a viable model of SUCLA2‐deficient mitochondrial myopathy has been achieved. This report therefore presents the initial investigations into adult murine skeletal muscular phenotypes of SUCLA2 deficiency. On the organismal level, research objectives included determining the whole‐body phenotypic effects of the muscle‐specific KO of *Sucla2*, including assessment of growth, strength and movement, to determine the role of skeletal muscle as a driver of disease. This work also sought to explore the functional consequences of SUCLA2 loss on the tissue, cellular and molecular levels within two distinct, yet similarly sized muscles within the murine hindlimb: the primarily glycolytic extensor digitorum longus (EDL) and the more oxidative soleus (SOL) muscle. Importantly, our tissue‐specific model allows us to determine the direct effect of SCS loss on skeletal muscles without the possibly confounding effect of SCS loss in motor neurons. In summary, this work presents an in vivo mouse model, providing a vital tool for mitochondrial myopathy and underscoring the important opportunities for investigation of myopathic mechanisms on the tissue and muscle fibre levels.

## 2 Methods

### 2.1 Animals

All experiments were approved by the Institutional Animal Care and Use Committee (IACUC) at Indiana University. Mice were bred at Indiana University School of Medicine and maintained in a 12/12‐h light/dark cycle. Room temperatures are maintained at 19°C–24°C with 40%–60% humidity. All mice were housed in positive‐pressure, individually ventilated cages. Standard autoclaved 6% fat diet (Purina Lab Diet 5K52) was available to the mice ad libitum, as was water with acidity regulated from pH 2.5 to 3.0. Wet feed was provided in the bottom of all cages to ensure the survival of *Sucla2* mutant mice. The *Sucla2* floxed mouse line was generated as described [18], and the HSA‐Cre transgenic line was obtained from Jackson Laboratories [17].

### 2.2 Genotyping

DNA was purified from mouse tail snips via ethanol precipitation. *Sucla2* genotypes were determined as described [18]. Primer sequences are included within the supplemental methods. The presence or absence of the Cre recombinase was determined as published [17].

### 2.3 qPCR and RT‐qPCR

Relative mtDNA content was measured using real‐time TaqMan qPCR as described [18, 19].

Total RNA was isolated from newborn mouse hindlimb muscle using the Quick‐RNA Miniprep Plus system, and cDNA was prepared using the GoScript Reverse Transcriptase System. Predesigned TaqMan assays for SCS components were run on duplexed TaqMan qPCR with an assay for *Gapdh* as published [18].

### 2.4 Measurement of Serum Metabolites

Serum amino acids, methylmalonic acid, and acylcarnitines were analysed using stable isotope dilution liquid chromatography–tandem mass spectrometry (LC–MS/MS). For amino acid analyses, clarified supernatant was analysed using a biphasic LC–MS/MS approach first with a HILIC separation [20] and next using a mixed‐mode chromatographic separation [21]. Methylmalonic acid quantification was completed following the method described by Pedersen et al. [22], and the measurement and quantification of acylcarnitines was completed as described by Luna et al. [23].

Serum concentrations of lactate were measured via colorimetric enzyme assay kits according to manufacturer's protocols (Sigma‐Aldrich: MAK064). Serum concentrations of FGF‐21 (Abcam: ab212160) were measured via ELISA.

Adenine nucleotides were measured on methanol extracts using ultra performance liquid chromatography as done previously [24]. Guanine nucleotides were measured using the same chromatography method, but extracts were first derivatized with 8‐(diazo methyl) quinoline [25].

### 2.5 Measurement of SCS Enzyme Activity

SCS activity was measured in whole‐cell lysates from murine skeletal muscle tissues as described [18].

### 2.6 Western Blotting

Whole‐cell lysates of mouse skeletal muscle were prepared in standard RIPA buffer. Total protein concentrations were determined via the Pierce BCA Protein Assay. Gel electrophoresis, and western blotting were conducted as described [18]. All antibody conditions are provided in the Supporting Information.

### 2.7 Gait Analysis

Animal‐safe ink was used to determine the location of the mouse footpads on standard 8.5 in × 11 in printer paper for at least four consecutive steps. Forelimb and hindlimb stride length and width were measured from the centre of the mouse footpad as demonstrated in Figure S1 and as described by Wertman et al. [26].

### 2.8 Grip Strength Measurement

Testing apparatus (BioSeb BIO‐GS3 Grip Strength Meter) was a rectangular metal grid attached to a T‐shaped sensor bar, used to measure the maximum force an animal exerts just prior to releasing its grip. Forelimb strength was tested by lowering the mice onto the testing grid so that their front paws grasped the mesh near the crossbar. Hindlimb strength was tested by placing the mice onto the grid with all four paws below the crossbar. Each mouse's tail was gripped near the base and lifted towards the ceiling so that the hind limbs released their grip on the grid, but the forelimbs remained. In both cases, the force required to cause this release was recorded twice.

### 2.9 Open Field Testing

Mice were observed for 30 min. The testing apparatus is a square arena enclosed in a darkened, sound‐dampening chamber. Each of the open field arenas is surrounded by a frame housing 48 photobeams to track movement in three dimensions. The x‐axis and y‐axis sensors were placed approximately 1 cm above the floor of the arena, and the z‐axis sensors were placed at approximately 6 cm. Each arena contains an opaque shelter occupying half of the total area, with an opening in the centre at ground level for access. All mice were placed in the arena inside the darkened shelter and allowed to freely explore once the environmental chamber door was closed. Photobeams tracking the animal on the x‐, y‐, and z‐axes enabled quantification of distance, total movement time, vertical movement and position.

### 2.10 Running Wheel Behaviour and Food/Water Consumption

Testing apparatus was a TSE Phenomaster Home‐Cage Telemetry System, which included a 33 cm × 18 cm rectangular cage with corncob bedding, a 12‐cm diameter running wheel, food measurement system and two drink measurement systems. Running wheel activity and food and water consumption were recorded every 15 min by telemetry software for 10 days. Individually caged mice had unrestricted access to the wheel, food, water and nest material.

### 2.11 Ex Vivo Muscle Contractions

Mice were anesthetized using a ketamine (90 mg/kg) and xylazine (10 mg/kg) cocktail, delivered intraperitoneally and were humanely euthanized following tissue collection. Supplemental oxygen was provided by nosecone. EDL and SOL muscle isolations were performed as described [27]. The muscles were immediately transferred to Krebs–Henseleit Buffer (25‐mM NaHCO_3_, 118‐mM NaCl, 4.7‐mM KCl, 1.2‐mM MgSO_4_, 1.2‐mM KH_2_PO_4_, 1.2‐mM CaCl_2_) with added glucose (5 mM) and sodium pyruvate (0.15 mM).

Muscles were suspended vertically in a 37°C tissue bath containing Krebs–Henseleit buffer solution and gassed continuously with a 95% O_2_/5% CO_2_ mixture to a pH of 7.4. Muscles were suspended at a resting tension of ~8 mN for 10 min. Optimal length (Lo) was determined as described [27] and used to estimate muscle cross‐sectional area (CSA) for normalization of muscle raw force (Po) to specific force (sPo). Approximately 5 min after confirming optimal length and maximal twitch force, the muscles were stimulated every 3 min with increasing pulse frequencies. The muscles were then allowed to rest for 5 min before subjecting the muscles to muscle‐specific fatiguing contractions, as published [27]. Respective frequencies for both experiments are provided in the Supporting Information.

### 2.12 Myosin Heavy Chain Immunofluorescence and Histochemical Staining

SOL and EDL muscles from *Sucla2* mutants and control mice were extracted and frozen in liquid nitrogen‐cooled isopentane. Muscles were embedded in O.C.T (Tissue‐Tek: 4583) and stored at −80°C until cryosectioning on a Leica CM 1950 Cryostat at −21°C. Ten micrometres of cross sections were permeabilized with 1X PBS 0.1% Triton‐X100, blocked with 0.5% BSA and 10% goat serum in 1X PBS for 1 h. Primary antibodies included those for laminin, MyHC–slow, MyHC 2A and MyHC 2B. After an overnight incubation, the slides were washed with 1X PBS and probed with the secondary antibodies for 1 h. Conditions for all antibody incubations are provided in the Supporting Information. Then, the slides were mounted with Diamond Antifade Mountant, covered with a coverslip and sealed.

The imaging was performed with a Keyence BZ‐X810 microscope system from the Indiana Center for Biological Microscopy (ICBM). Entire muscle sections were scanned using a 10× objective, and the resulting images were stitched using the BZ‐X800E Analyzer. To analyse the images, the montage files were converted for each channel into 8‐bit using ImageJ. The CSA and typification of myofibres were determined using QuantiMus [28].

Additional cross sections from the same muscles were used for histochemical staining for cytochrome *c* oxidase (COX), succinate dehydrogenase (SDH) and lipids via oil red O (ORO). For COX staining, slides were treated with 1 mL of a medium containing 15 mg of 3,3‐diaminobenzidine tetrahydrochloride (DAB), 30 mg of cytochrome *c*, 3 mg of bovine catalase and 2.25 g of sucrose in 50‐mM phosphate buffer, pH 7.4 for 90 min at 37°C. Slides were then rinsed with distilled water and treated with Baker's calcium formalin for 10–15 min. Slides were rinsed with water and dehydrated before imaging. An orange/brown chromogen deposit indicates COX activity.

For SHD staining, slides were treated with 1 mL of medium containing 0.2 M of sodium succinate, 0.2 M of phosphate buffer, nitro blue tetrazolium and hydrochloric acid for 90 min at 37°C. Slides were rinsed before a 10‐ to 15‐min treatment with Baker's calcium formalin. A blue/purple formazan deposit forms at the site of SDH activity viewed at imaging.

For ORO staining, slides are first fixed in Baker's formol calcium, before rinsed and incubated in 0.5% ORO stock solution in isopropyl alcohol for 10–15 min at room temperature. Slides were then rinsed in water for 20–30 min and stained with haematoxylin for 30 s. Nuclei are stained blue, and fat is stained red. Slides for all three staining procedures were imaged on a Leica DM300 LED binocular microscope.

### 2.13 Transmission Electron Microscopy

The specimens were fixed in 3% Glutaraldehyde/0.1 M cacodylate buffer. Then, specimens were rinsed with buffer followed by post‐fixation with 1% osmium tetroxide in cacodylate buffer for 1 h. After rinsing again, the tissue specimens were dehydrated through a series of graded ethyl alcohols from 70% to 100%. The specimens were then infiltrated with two changes of 100% acetone and a 50:50 mixture of acetone and embedding resin for 3 days. The acetone was then allowed to evaporate for 3 h. Then, specimens were embedded in a fresh charge of 100% embedding media. Following polymerization overnight at 60°C, 80‐ to 90‐mm sections were cut from the blocks, stained with uranyl acetate and viewed on a Tecnai Spirit with digital images taken with an Advanced Microscope Techniques CCD camera.

### 2.14 Statistical Analysis

Data are represented as means ± standard deviations. Statistical significance was set at a *p*‐value of < 0.05. In all experiments, the sample size (*n*) represents the number of biological replicates per experimental group and is also provided within the corresponding legends. All datasets were subject to a Shapiro–Wilk test for normalization and Grubb's outlier test with an alpha cutoff of 0.05, with any identified outliers removed from the dataset. Two‐tailed unpaired *t*‐tests were applied when comparing single measures between two groups, and multiple standard unpaired *t*‐tests were applied to grouped analyses with variables measured within single experiments. False discovery rate (FDR) method was applied for multiple testing corrections. For data comparing three or more groups, two‐way ANOVA was performed followed by a Šídák post hoc test for discoveries. Statistical analysis for longitudinal studies included calculation of areas under the curve (AUC) with subsequent *t*‐test comparisons, and effects of two‐dimensional X‐Y data were assessed via mixed‐effects model. The statistical tests performed for each dataset are indicated within the figure legends, and all statistical analyses were performed using GraphPad Prism version 9.1.2.

## 3 Results

### 3.1 Molecular Validation of Skeletal Muscle *Sucla2* Deficiency Reveals Biochemical Markers of Mitochondrial Dysfunction

**FIGURE 1:**
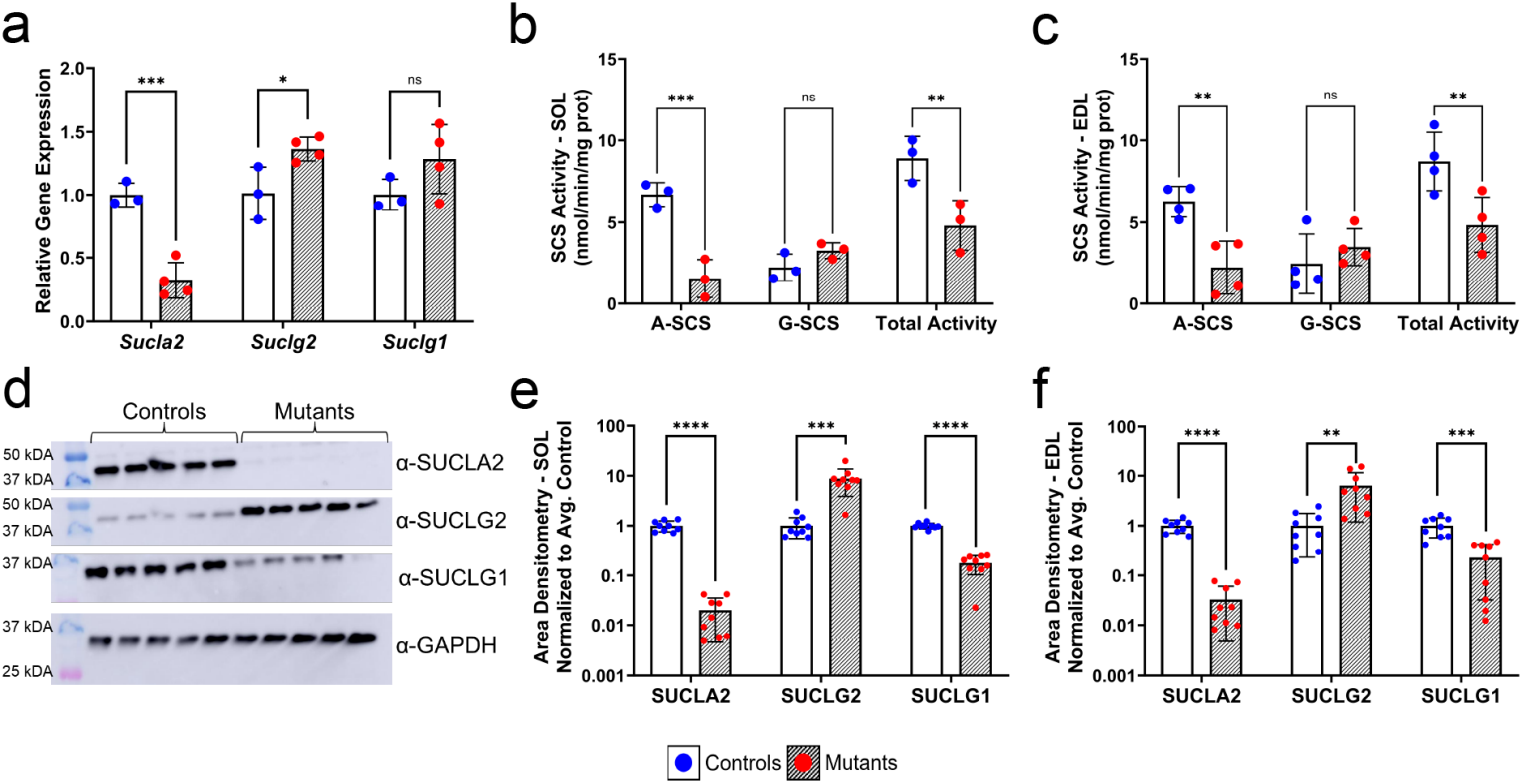
Functional variants in *Sucla2* lead to unique patterns of SCS expression and activity in skeletal muscle tissues. (a) Relative gene expression of SCS components measured via RT‐qPCR in hindlimb muscles of *Sucla2* wild‐type (WT) mice (blue) and conditional knock‐out (KO) mice (red) at postnatal day zero (p0), *n* = 3–4. (b–c) ADP‐ and GDP‐specific enzymatic activity of SCS in whole‐cell lysates of the (b) soleus (SOL), *n* = 3, and (c) extensor digitorum longus (EDL), *n* = 4, of adult mice. Total activity is defined as the sum of nucleotide‐specific activities (Table S3). (d) Composite image of western blot analyses of SCS components in the SOL (d) and EDL (Table S4). (e–f) Densitometry quantitation calculated using ImageJ software in both the SOL (e) and EDL (f), *n* = 9. Densitometry results are plotted on a logarithmic axis. All data presented in bar graphs are represented as means ± SD. For all data shown, the WT (controls) genotype *Sucla2* ^*+/+*^, *HSA*‐Cre positive, and KO (mutants) mice are either *Sucla2* ^*−/−*^, *HSA*‐Cre positive. *Sucla2* alleles and equal levels of KO between the *Sucla2* mutant genotypes are outlined in Figure S2. Significant differences are depicted by asterisks, where * = *p* < 0.05, ** = *p* < 0.01, *** = *p* < 0.001 and **** = *p* < 0.0001 by multiple unpaired *t*‐tests, with *p*‐values corrected for multiple testing via false discovery rate (FDR).

KO of *Sucla2* within skeletal muscle was achieved through Cre‐recombination of a CRISPR‐Cas9–generated *Sucla2* floxed allele, driven by the human skeletal actin (*HSA*) promoter [29]. Additionally, a constitutive deletion allele was recovered as a byproduct of the CRISPR event via nonhomologous end‐joining DNA repair. *Sucla2* mutant (*Sucla2* ^*−/−*^) mice within this study were either homozygous for the floxed allele or compound heterozygous for the floxed allele and the constitutive *Sucla2* deletion. Diagrams of all *Sucla2* alleles used and demonstration of equivalent KO between mutant genotypes in Cre‐positive animals are provided in Figure S2. RT‐qPCR analysis demonstrated 68% reduced *Sucla2* gene expression in the hindlimb muscles of mice at postnatal day zero (p0), with reciprocal increase in the alternative beta isoform of SCS (*Suclg2*) (Figure 1a). However, no difference was seen in the mRNA expression of the SCS alpha subunit, *Suclg1*.

Enzyme activity analysis of hindlimb muscles in 20–30‐week‐old mice confirmed functional *Sucla2* KO in adulthood. In the mutant SOL, the ADP‐specific SCS activity is ~23% of that of controls with no significant change in GDP‐specific activity (Figure 1b and Table S3). This pattern of enzymatic activity is also seen in the EDL (Figure 1c and Table S3), with roughly 55% residual total SCS activity in both tissues of *Sucla2* ^−/−^ mice when compared with that of WT controls. In both the SOL and EDL hindlimb muscles, we see greater than 95% reduction in SUCLA2 protein levels in the mutants (Figure 1d–f and Table S4) and a concomitant sevenfold increase in SUCLG2 protein expression in the SOL (Figure 1d–e). However, the catalytic alpha subunit, SUCLG1, is about 70%–80% posttranscriptionally downregulated. Similar patterns are observed in the EDL (Figure 1f and Table S4).

**FIGURE 2:**
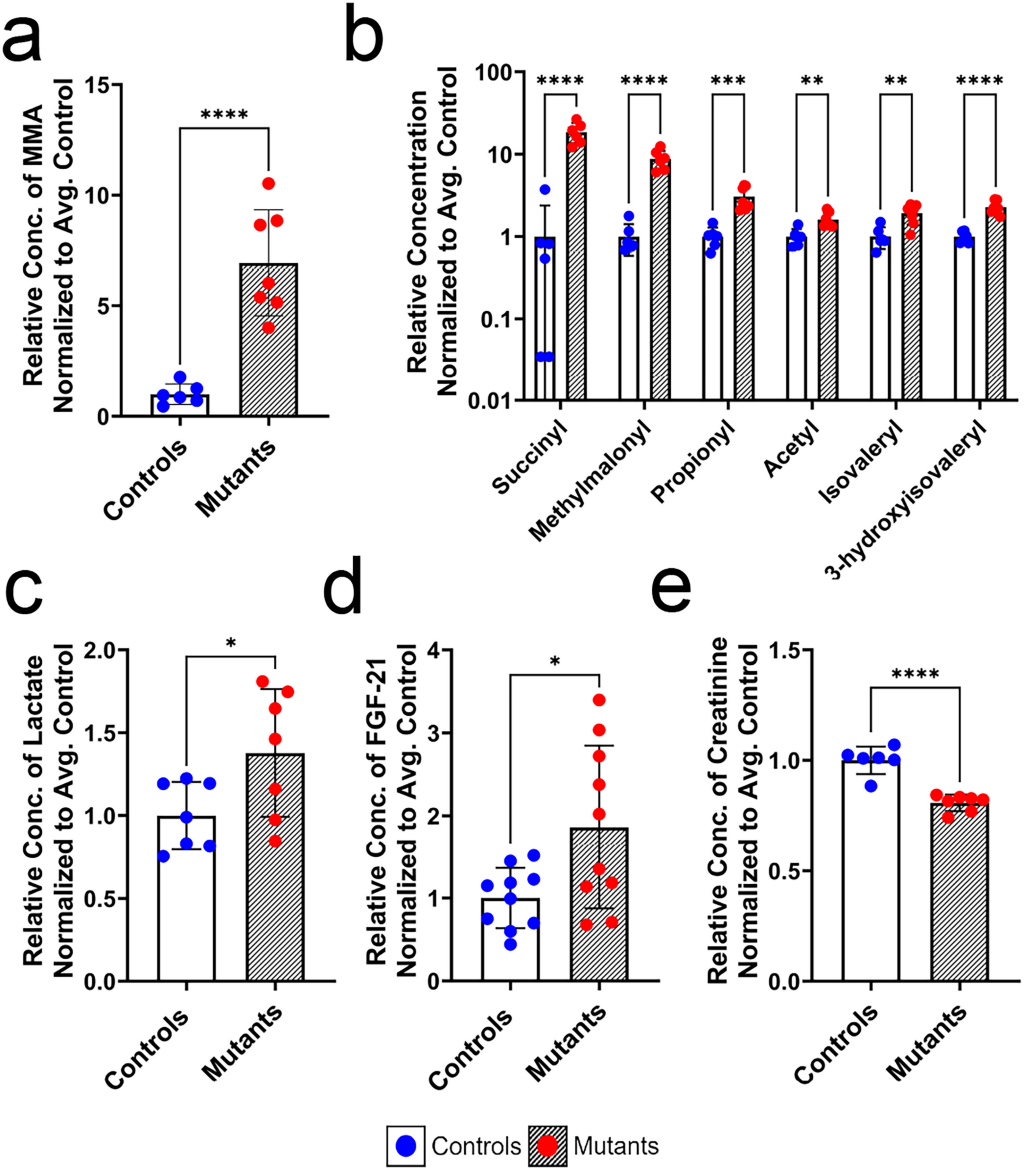
Metabolic markers of myopathy are observed in the serum of mice deficient for SUCLA2 in the skeletal muscle. (a) Serum levels of methylmalonic acid, *n* = 6–7. (b) Serum levels of significantly altered acylcarnitines (Dataset S1), *n* = 6–7. (c) Serum concentrations of lactate, *n* = 7. (d) Serum concentrations of FGF‐21, *n* = 10. (e) Serum concentrations of creatinine, *n* = 6–7. All data are presented as means ± SD and normalized to the average control. For all data shown, the control genotype is *Sucla2* ^*+/+*^, *HSA*‐Cre positive, and mutant mice are *Sucla2* ^*−/−*^, *HSA*‐Cre positive. Significant differences are depicted by asterisks, where * = *p* < 0.05, ** = *p* < 0.01, *** = *p* < 0.001 and **** = *p* < 0.0001 by standard unpaired *t*‐tests. Figure (b) depicts only acylcarnitines species that were significantly different between genotypes following multiple unpaired *t*‐tests with FDR correction. All measured metabolites are provided in Dataset S1.

Serum metabolomics was performed to investigate SCS‐deficient metabolic dysfunction. Diagnostic markers of SCS deficiency, including a nearly sevenfold increase in serum methylmalonic acid (Figure 2a), elevations in various acylcarnitines (Figure 2b), and a slight (37%) increase in serum lactate (Figure 2c), were observed, supporting a metabolic block within the TCA cycle. Additionally, although no significant differences were seen in creatine kinase activity or serum levels of growth and differentiation factor 15 (GDF‐15) (Figure S3a–b), there was nearly a twofold increase in serum levels of the myokine fibroblast growth factor 21 (FGF‐21) (Figure 2d), as well as a significant reduction (20%) in serum creatinine (Figure 2e) in the *Sucla2* deficient mice, suggestive of both mitochondrial dysfunction and reduced muscle mass [30, 31].

Furthermore, the observed increase in acylcarnitines, particularly the 18‐fold increase in succinyl‐carnitine and threefold increase in propionyl‐carnitine (Figure 2b), in conjunction with the perturbed SCS function, is likely indicative of a cellular accumulation of their cognate acyl‐CoA species. Acyl‐CoA molecules are substrates for posttranslational protein acylation, which has been linked to pathogenesis in other models of SCS deficiency [14, 18]. Increases in both protein succinylation and propionylation were observed in the SOL and EDL muscles of *Sucla2* KO mice (Figure S3c–d). Altogether, the metabolic profile supports the presence of significant TCA cycle perturbation with similarities in both SOL and EDL.

### 3.2 KO of *Sucla2* Within the Murine Skeletal Muscle Results in Reduced Muscle Strength and Activity

**FIGURE 3:**
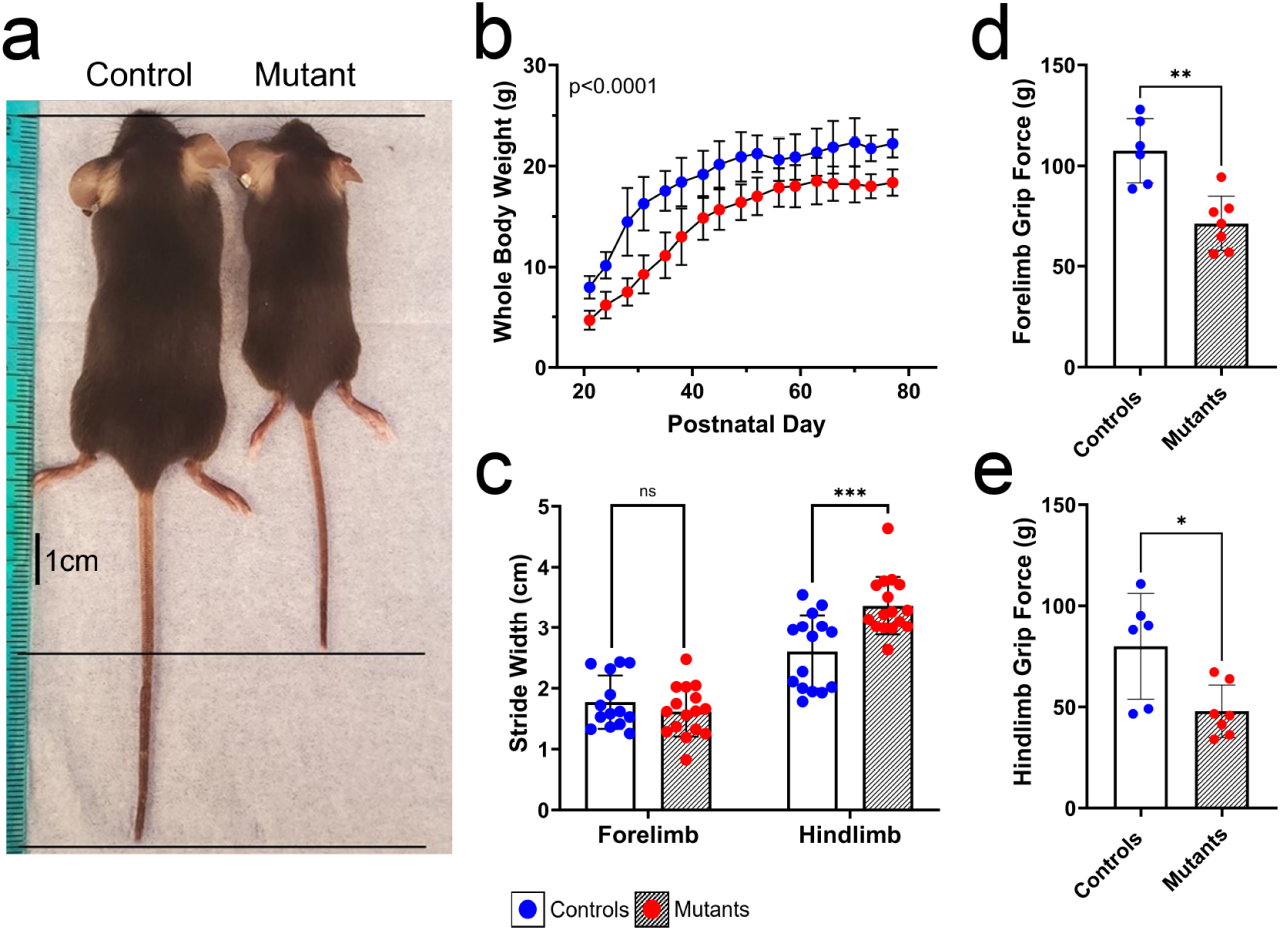
Skeletal muscle‐specific *Sucla2* deficiency yields an in vivo model of myopathy with significant growth deficiency. (a) Representative image depicting WT and KO female mice at p21. (b) Body weights of *Sucla2* WT and KO mice from p21 to p77 (3 to 11 weeks of age), *n* = 21. *p*‐value indicates statistically significant effects of both genotype and time via mixed‐effects model. (c) Gait analysis of mice at p21, *n* = 4 consecutive steps of four mice per genotype, and stride length (data not shown, not significant) and (c) stride width of both forelimbs and hindlimbs were measured. (d–e) Two‐paw grip strength measured on both forelimb (d) and hindlimb paws, *n* = 7–8. All measured behavioural phenotypes are provided in Dataset S2 and Figure S4. All data are presented as means ± SD, and significant differences are depicted by asterisks, where * = *p* < 0.05, ** = *p* < 0.01 and *** = *p* < 0.001 by standard unpaired *t*‐tests, with a *p*‐value correction via FDR calculated for the gait analysis. For all data shown, the control genotype is *Sucla2* ^*+/+*^, *HSA*‐Cre positive, and Mutant mice are *Sucla2* ^*−/−*^, *HSA*‐Cre positive.

**FIGURE 4:**
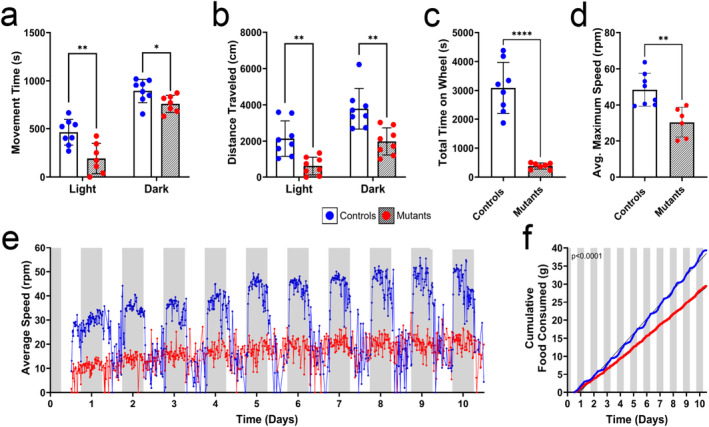
*Sucla2* skeletal muscle‐specific knock‐out mice exhibit reduced physical activity. Mice were observed in an open field with access to both a light and dark area. (a) Total movement time and (b) distance travelled were measured in both the light and dark environments, *n* = 7–8. Mice were housed in a telemetry cage with unrestricted access to a running wheel, food and water for approximately 10 days, and running wheel activity (c–e) and feeding behaviour (f) were monitored, *n* = 7–8. For figures (e)–(f), the white and grey columns indicate alternating 12‐h periods of light and dark, respectively. All data presented in bar graphs are means ± SD, and significant differences are depicted by asterisks, where * = *p* < 0.05, ** = *p* < 0.01 and **** = *p* < 0.0001 by multiple unpaired *t*‐tests with *p*‐value correction for multiple testing via FDR where necessary. Statistical comparison of longitudinal data was conducted by *t*‐test comparisons following area under the curve (AUC) analysis, and consumption behaviour was conducted via linear regression analysis, with the *p*‐value representing significantly differing slopes. For all data shown, the WT genotype is *Sucla2* ^*+/+*^, *HSA*‐Cre positive, and KO mice are *Sucla2* ^*−/−*^, *HSA*‐Cre positive. All behavioural data measured are shown in Figure S4 with raw data provided in Dataset S2.

Whole‐body phenotypes of *Sucla2* mutant and control mice were followed from 3 to 14 weeks of age. A representative image of the size difference between *Sucla2* ^*−/−*^ and control mice at 3 weeks of age is provided in Figure 3a, and the growth of 21 mice per genotype, with equal proportions of males and females, was recorded from 3 to 11 weeks of age. At weaning (p21), *Sucla2* KO mice were 44% the size of WT age‐matched controls and remained smaller throughout young adulthood (*p* < 0.0001) (Figure 3b). Preliminary observations revealed a widened hindlimb gait (*p* < 0.05) (Figure 3c) and a 34% to 40% reduction in grip strength in both the forelimbs (Figure 3d) and hindlimbs (Figure 3e) of SUCLA2‐deficient mice, respectively. Mice were subject to open field analysis, with access to both a light and dark environment, where *Sucla2* mutant mice spent half as much time moving within the lighted chamber as WT controls (Figure 4a), and the distance travelled by *Sucla2* KO mice was reduced compared with that by controls in both environments, with mutant mice moving about 67% less in the light and 44% less in the dark (Figure 4b).

**FIGURE 5:**
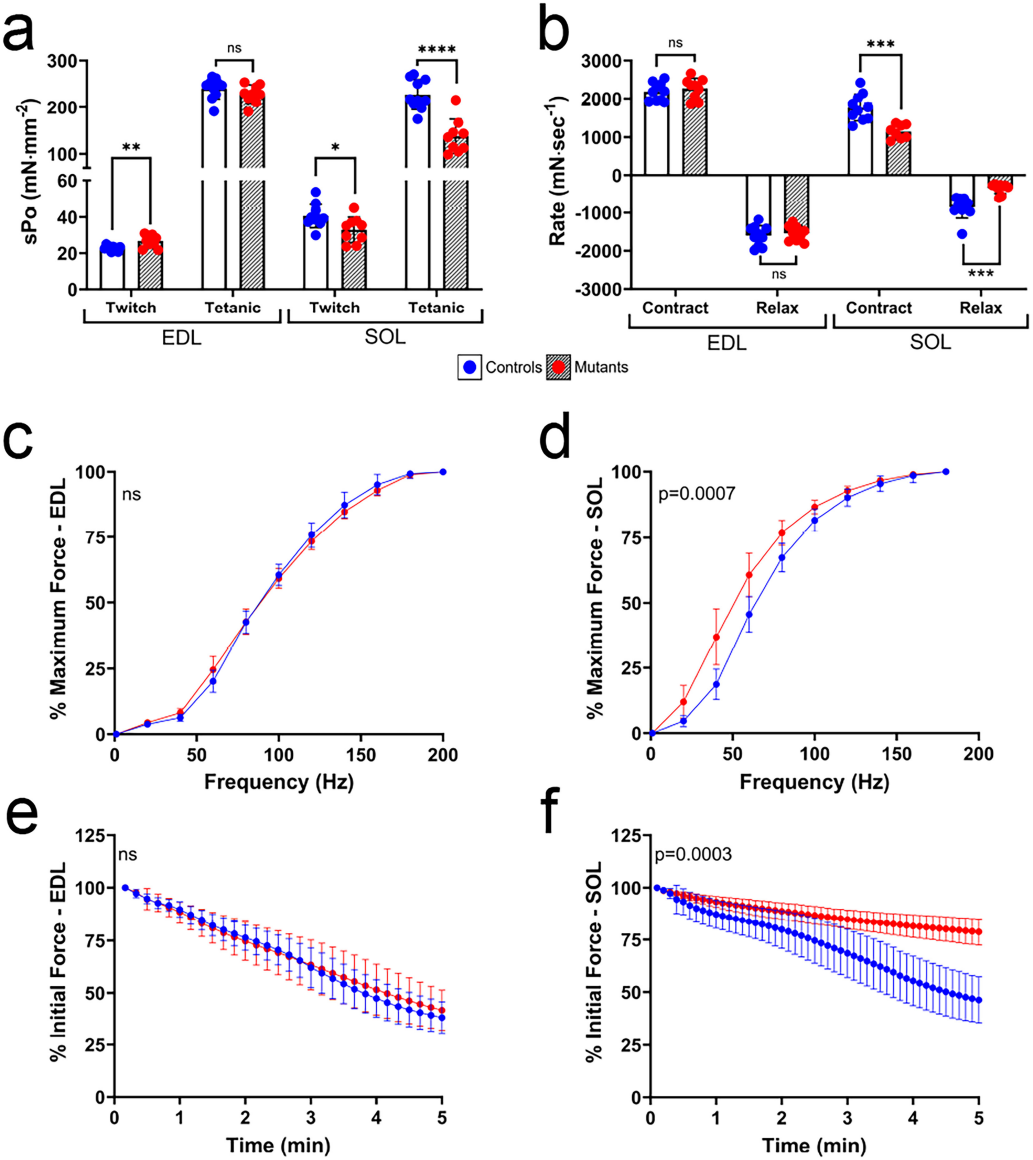
SUCLA2 loss in skeletal muscle creates different phenotypic effects on slow‐ and fast‐twitch muscles. (a) Twitch and tetanic specific force and (b) the rate of muscle contraction and relaxation of the SOL and EDL were measured ex vivo. The data are presented as means ± SD, and statistically significant differences are depicted by asterisks, where * = *p* < 0.05, ** = *p* < 0.02, *** = *p* < 0.001 and **** = *p* < 0.0001 by multiple unpaired *t*‐tests, with *p*‐values corrected via FDR. (c–d) The relationships between frequency of stimulus and resultant force recorded in the (c) EDL and (d) SOL of WT (blue) and KO (red) mice. (e–f) The force measured in response to a repeated pulse frequency over 5 min to assess muscle fatigue in the EDL (e) and SOL (f). For figures (c)–(f), *p*‐values indicate a statistically significant effect of genotype via a mixed‐effects model. For all data shown, the WT genotype is *Sucla2* ^*+/+*^, *HSA*‐Cre positive, and KO mice are *Sucla2* ^*−/−*^, *HSA*‐Cre positive. All ex vivo muscle function data are provided in Dataset S3, *n* = 9–10.

To further investigate locomotor activity, mice were housed in telemetry cages with unrestricted access to food, water and a running wheel. Mice deficient for SUCLA2 within the skeletal muscle spent about 88% less cumulative time on the running wheel than that of WT controls (Figure 4c). When the mice were on the wheel, they ran less. The average maximum speed reached by *Sucla2* mutant mice was nearly 40% lower than the average maximum speed of control mice (Figure 4d), and a longitudinal map of the average wheel speed over 14 days, provided in Figure 5e, clearly illustrates the overall decreased running speed of the skeletal muscle KO mice over time, with over 126 000 less wheel rotations than that of controls on average (Figure S4f). The complete dataset collected during whole animal phenotyping is provided in Figure S4 and Dataset S2. Food and water consumption were also analysed, and the rates of consumption of both food (Figure 4f) and water (Figure S4e) in the Sucla2^−/−^ mice were significantly reduced (*p* < 0.0001). However, upon normalizing the consumption data to body weights, the differences in consumption were minimized (Figure S5). Interestingly, the standard circadian pattern of nocturnal consumption within the controls is lost in the SUCLA2‐deficient mice for both food and water intake (Figure 4f and Figure S4e). Taken together, the whole‐body behavioural phenotypic patterns provide significant support for a SUCLA2‐deficient model with reduced growth, muscle strength and spontaneous locomotor activity in vivo.

### 3.3 Loss of SUCLA2 Produces Distinct Phenotypes Within the EDL and SOL Muscles of the Hindlimb

To explore muscle contractile function directly, the SOL and EDL muscles were subject to ex vivo isometric contraction studies (all data provided in Dataset S3). Although the SUCLA2‐deficient EDL muscles showed a ~15% greater (*p* < 0.01) specific twitch force than that of WT controls, the SUCLA2‐deficient SOL muscles showed a 19% lower specific twitch force (*p* < 0.05) (Figure 5a), where specific force is force normalized to the physiological CSA. Furthermore, a substantial reduction (~40%) in tetanic specific force was observed in the mutant SOL, whereas no differences in maximal force between genotypes were observed in the EDL (Figure 5a). Additionally, although no significant genotype‐specific differences in contractile kinetics were observed within the EDL, the SUCLA2‐deficient SOL muscles demonstrated 35% slower rates of contraction and 60% slower rates of relaxation (Figure 5b).

**FIGURE 6:**
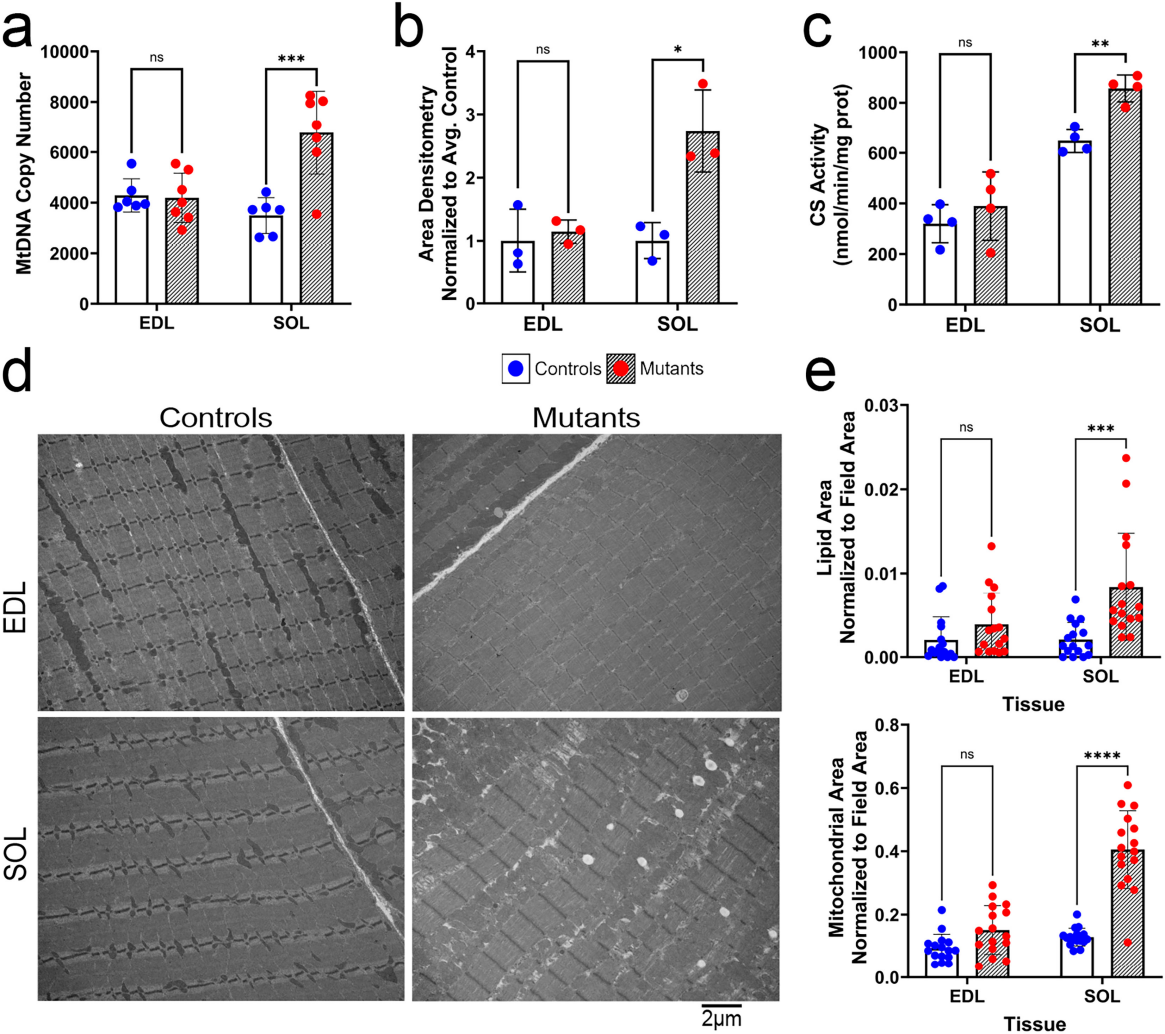
Evidence for mitochondrial proliferation is observed in SUCLA2‐deficient SOL. (a) The copy number of the mitochondrial chromosome (mtDNA) measured via qPCR in the EDL and SOL, *n* = 6–7. (b) Densitometry quantification of western blots against citrate synthase conducted using ImageJ in the EDL and SOL, *n* = 3. (c) Citrate synthase enzymatic activity in the EDL and SOL, *n* = 4. (d) Representative transmission electron microscopy (TEM) images taken of the EDL and SOL. For both muscles, the representative images were taken at a magnification of 6800×, scale bar = 2 μm. (e) ImageJ software was used to quantify the total lipid (top) and mitochondrial (bottom) area of *Sucla2* WT and KO mice of representative TEM images (*n* = 4 images per mouse, 4 mice per genotype). All TEM quantification data are provided in Table S6. Data in bar graphs are means ± SD, and significant differences are depicted by asterisks, where * = *p* < 0.05, ** = *p* < 0.01, *** = *p* < 0.001 and **** = *p* < 0.0001 by multiple unpaired *t*‐tests with *p*‐values corrected via FDR. For all data shown, the WT genotype is *Sucla2* ^*+/+*^, *HSA*‐Cre positive, and KO mice are *Sucla2* ^*−/−*^, *HSA*‐Cre positive.

The relationship between stimulus frequency and contractile force was measured by increasing the frequency of the electrical pulse stimulus. EDL muscles of the *Sucla2* KO and WT mice showed virtually identical force–frequency relationships, reaching 50% maximum force at ~96 Hz (Figure 5c). Conversely, in the SOL, there was a significant genotype‐specific difference (*p* < 0.0001) in the force–frequency relationships, with a leftward shift of the mutant curve; although 65 Hz was required in the WT mice, the *Sucla2* KO mice reached 50% maximum force at about 52 Hz on average (Figure 5d). Differences between muscle types were also observed with repeated stimulations designed to elicit fatigue. Figure 6e again shows identical curves between genotypes in the EDL, with both decreasing to ~50% of initial force at 5 min. However, the SUCLA2‐deficient SOL is significantly fatigue‐resistant compared with control (Figure 5f), maintaining over 75% of the initial contractile force at the end of the stimulation period.

**FIGURE 7:**
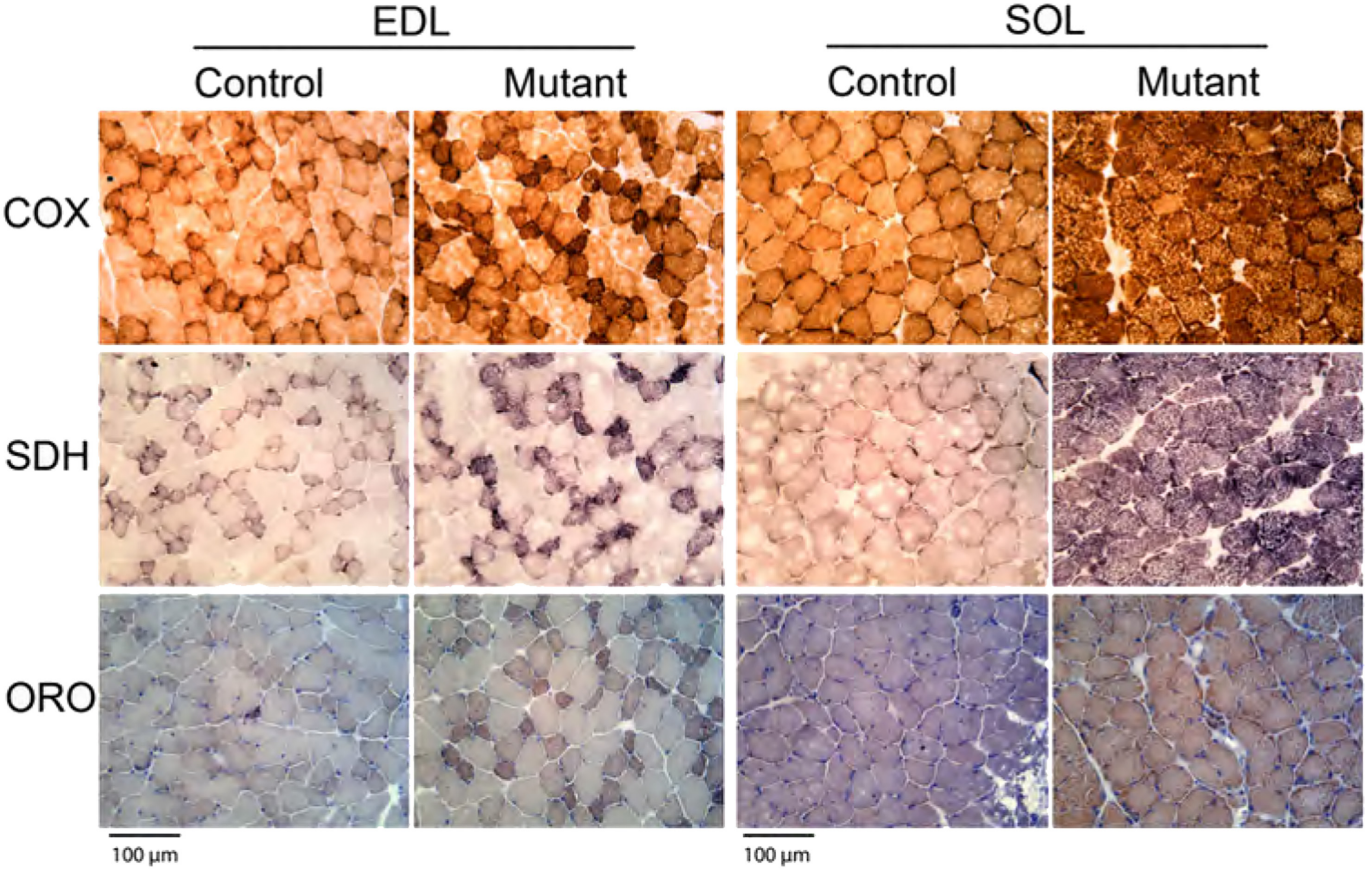
Histochemical staining of mitochondrial enzymes shows different levels of mitochondrial proliferation in skeletal muscles. Histochemical staining of mitochondrial cytochrome *c* oxidase (COX) and succinate dehydrogenase (SDH), and lipid staining via oil red O (ORO) in WT and KO mice in the EDL and SOL. All images were taken at 40× magnification, scale bar = 100 μm. The WT genotype is *Sucla2* ^*+/+*^, *HSA*‐Cre positive, and KO mice are *Sucla2* ^*−/−*^, *HSA*‐Cre positive.

Differences between the EDL and SOL muscles in the *Sucla2* model were again observed at the cellular and molecular level, with increased markers of mitochondrial mass observed more prevalently in the mutant SOL. First, a 1.4‐fold (*p* < 0.001) increase in mitochondrial DNA (mtDNA) copy number was observed within the mutant SOL (Figure 6a). This was surprising, as SCS symptoms have been historically associated with mtDNA depletion. The traditional pathogenic mechanism positions SCS as a crucial player in maintaining the mitochondrial nucleotide pool via its physical interaction with mitochondrial nucleoside diphosphate kinase (NDPK‐M) [8]. However, there were no observed alterations in the gene encoding for NDPK‐M, and no differences were observed in whole‐cell concentrations of adenine or guanine nucleotides (Figure S7). Evidence for mitochondrial proliferation was further supported by an increase in the protein and activity level of TCA cycle enzyme citrate synthase in SUCLA2‐deficient SOL, with no differences observed between genotypes in the EDL (Figure 6b–c). Transmission electron microscopy (TEM) images were taken (Figure 6d), and although there were trends towards increased lipid (*p* = 0.37) and mitochondrial (*p* = 0.08) content within the mutant EDL, significantly (*p* < 0.001) greater lipid droplet (fourfold) and mitochondrial content (threefold) were observed within the SUCLA2‐deficient SOL compared with controls (Figure 6e and Table S6). Evidence for increased mitochondrial and lipid content within the SUCLA2‐deficient SOL is further demonstrated histochemically by markedly increased staining for respiratory enzymes COX and SDH, as well as increased lipid staining by ORO in the SOL with modestly observable increases in the EDL (Figure 7).

**FIGURE 8:**
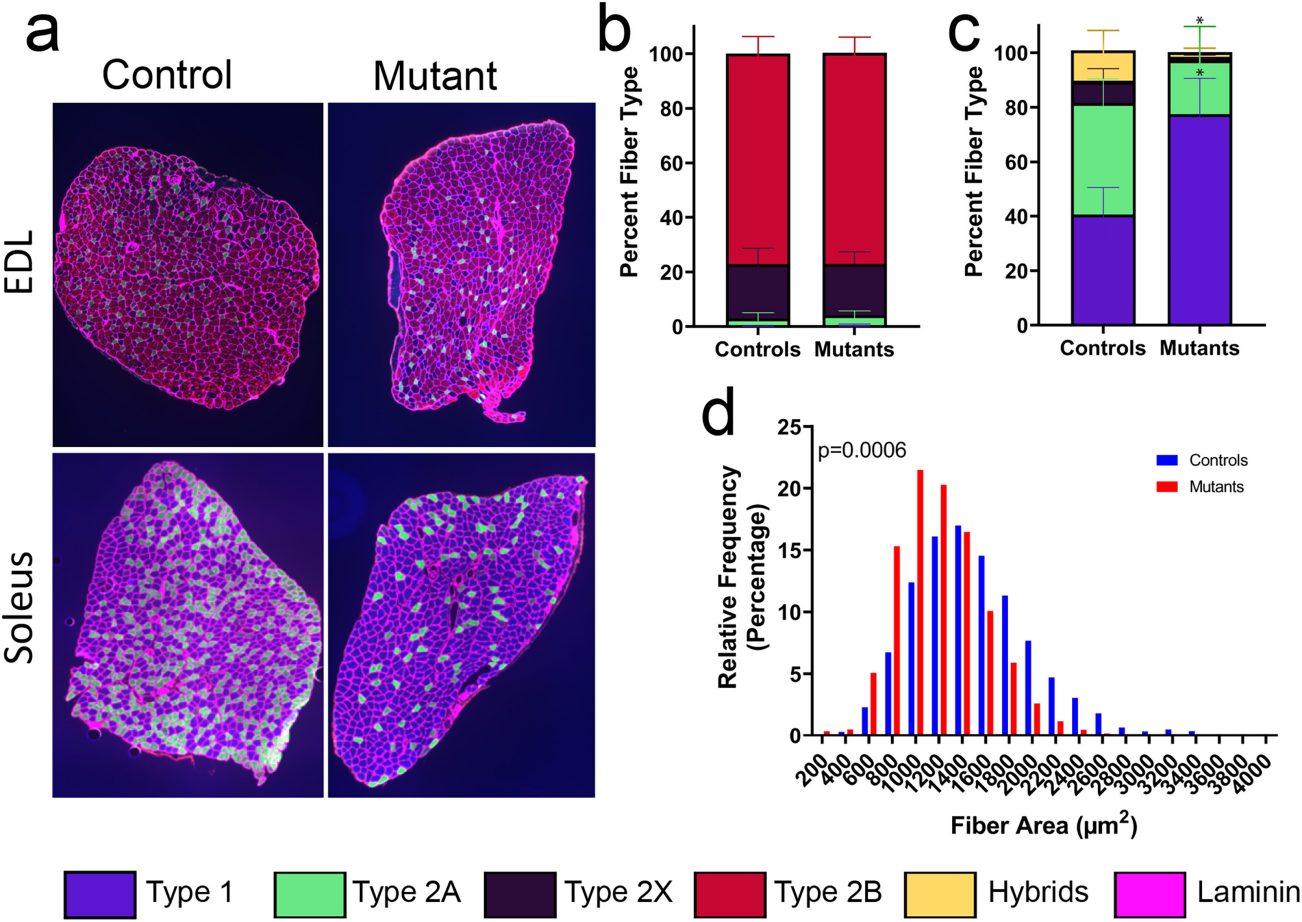
Loss of SUCLA2 in the skeletal muscle leads to muscle‐specific fibre‐type switching and Type 1 fibre predominance in the SOL. (a) Immunofluorescent staining for myosin heavy chains (MyHC) conducted to classify fibre‐type distribution in the EDL and SOL. (b–c) Percent fibre‐type contributions to the total surface area of the muscle sections calculated for both the (b) EDL (*n* = 6 animals per genotype, totalling 3407 fibres in WT and 4.894 fibres in KO). and (c) SOL (*n* = 6 animals per genotype, totalling 3603 fibres in WT and 5595 fibres in KO). The colour legend for both the staining and bar graphs is included at the bottom of the figure. Statistical differences between the distributions of fibre‐type percentages were calculated via two‐way ANOVA, with Šídák multiple comparisons test revealing statistically distinct proportions of Type 1 (*n* = 1538 fibres in WT and 4153 fibres in KO) and Type 2A (*n* = 1567 fibres in WT and 1117 fibres in KO) fibres within the SOL (*p < 0.0001), and no genotype‐specific changes of fibre proportions within the EDL. Individual analyses of all fibre types are provided in Figure S6. (d) The relative frequency of various cross‐sectional fibre areas (μm^2^) in the SOL of the *Sucla2* controls (blue) and mutants (red), with *p*‐value representing statistical differences between the distributions as calculated by Chi‐squared analysis. The WT genotype is *Sucla2* ^*+/+*^, *HSA*‐Cre positive, and KO mice are *Sucla2* ^*−/−*^, *HSA*‐Cre positive.

Given the observed changes, we next tested the hypothesis that muscle fibre–type proportions may differ between genotypes. Therefore, immunohistochemical staining was conducted for fibre type–specific myosin heavy chain isoforms (Figure 8a). In mutant EDL, there was an increase from 0.13% to 1.72% Type 1 fibres (Figure S5), but this change was small relative to proportions of other fibre types, such that overall fibre‐type area distribution was virtually unchanged between genotypes (*p* = 0.999) (Figure 8b). On the other hand, in the SOL, the proportion of Type 1 fibres nearly doubled from 40% to 78% within the SUCLA2‐deficient mice, with an accompanying proportional decrease in Type 2A fibres (*p* < 0.0001). Fibre type–specific quantification and statistical analyses for each muscle type are provided in Figure S6. Additionally, a leftward shift in the distribution of the cross‐sectional fibre area of the *Sucla2* mutant SOL was observed, indicating smaller fibres in the mutant (*p* < 0.001) (Figure 8d). Average fibre size in the SOL measured around 1209 ± 135 μm^2^ in the mutants versus 1461 ± 300 μm^2^ within WT controls (Figure 8d). Mean fibre size was not different in the EDL (1002 ± 174 μm^2^ in the WT vs. 985 ± 139 μm^2^ in the mutant) (Figure S6). These results yield further evidence for notably different cellular responses to SUCLA2 loss within the EDL and SOL that warrant further investigation.

## 4 Discussion

### 4.1 In Vivo and Molecular Phenotypes Provide Evidence for a Novel Model of Mitochondrial Myopathy With SUCLA2 Deficiency

This report presents a viable model of SUCLA2 deficiency with equivalent loss of the ADP‐specific isoform of SCS (A‐SCS) in both the SOL and EDL muscles of the mouse hindlimb (Figures 1 and 2). Skeletal muscle phenotypes are commonly observed in patients with SCS deficiency [3, 4, 7, 8, 9, 10, 11, 32]. The A‐SCS deficient mouse model presents with a severe observable myopathy, with mutant mice being noticeably smaller at weaning and throughout young adulthood (Figure 3a–b). *Sucla2* mutant mice exhibit significant muscle weakness in both the forelimbs and hindlimbs evidenced by lowered grip strength and a widened hindlimb gait (Figure 3c–e). Additionally, SUCLA2‐deficient mice exhibited decreased spontaneous movement patterns (Figures 4a–b and S4), including reduced exercise motivation and endurance when presented with a running wheel (Figures 4c–d and S4). This analysis was limited, however, in that the exercise was voluntary. Although this was valuable, future studies would benefit from induced physical exercise and tests of endurance to define endurance capacity more accurately.

Furthermore, although less food was consumed over time by the mutant mice, further analysis is required to assess the reasoning behind this observation. One likely explanation is the smaller size and reduced basal physical activity levels resulting from loss of SUCLA2, causing the mutant mice to require less food for energetic demands (Figure S4c), which is supported by the reduced differences in consumption data upon normalization to body weight (Figure S5). However, the loss of a circadian pattern of food consumption also points to abnormal consumption and may result in a metabolic deficit. Future investigation into perturbed metabolic pathways and function will address this hypothesis. Nonetheless, on the organismal level, SUCLA2‐deficient mice present with severe muscular phenotypes suggestive of mitochondrial myopathy, which is further evidenced by elevations of circulating FGF‐21, lower serum creatinine suggestive of reduced muscle mass, mitochondrial proliferation and Type 1 fibre predominance (Figures 2e–f, 7 and 8) [4, 28, 31, 33, 34].

SUCLA2‐deficient mitochondrial myopathy has been traditionally considered a mitochondrial DNA depletion syndrome (MDS) [7, 8, 9, 10, 11, 12]. This canonical hypothesis hinges upon evidence that SCS binds with the mitochondrial nucleotide diphosphate kinase (NDPK‐M), and loss of NDPK leads to impaired regulation of nucleotides essential for mtDNA replication [7, 8]. This mechanism is often associated with a loss of expression of *Nme4*, the gene encoding NDPK‐M or loss of NDPK‐M activity [35]; however, there were no observed differences in *Nme4* expression within the SOL or EDL in the present model (Figure S7). Additionally, whole‐cell concentrations of adenine and guanine nucleotides were assessed, and no differences between genotypes were noted in either tissue (Figure S7). Although the mitochondrial nucleotide pools were not measured specifically, this does point towards the lack of perturbed NDPK‐M activity, which most often facilitates phosphate transfer from ATP to GDP in mitochondrial nucleotide maintenance [35]. Furthermore, mtDNA depletion was notably absent within the presented model. Rather, an increase in mtDNA was observed specifically within the *Sucla2* mutant SOL muscle, in conjunction with increased citrate synthase activity, increased mitochondrial content imaged via TEM (Figure 6) and increased staining for mitochondrial enzymes SDH and COX (Figure 7). These results provide multiple layers of evidence for mitochondrial proliferation within the SUCLA2‐deficient SOL. Given these data, the muscle‐specific phenotypes presented do not seem dependent on NDPK‐M activity and mtDNA depletion.

Although symptoms of SUCLA2 deficiency have traditionally been linked to mtDNA depletion, there have been at least 10 reported cases of diagnosed, symptomatic SCS deficiency without mtDNA depletion in muscle in humans, suggesting that an alternative pathogenic mechanism exists [7]. More recent works in SCS research have recognized alterations in lysine acylation modifications to proteins as an alternative, or possibly additional, pathological mechanism leading to clinical manifestations of mitochondrial disease [7]. Specifically, altered protein succinylation caused by accumulation of succinyl‐CoA has been shown to contribute to respiratory deficiency independently of mtDNA depletion in models of SUCLA2 deficiency [14, 18]. Within the current work, the observed increase in serum concentrations of acylcarnitines (Figure 2a), and likely circulating levels of their cognate acyl‐CoA species, is accompanied by global increases in protein acylation modifications (Figure S3). Marked protein hypersuccinylation was observed in both the SOL and EDL upon SUCLA2 loss. Importantly, succinylation of myosin heavy chain reduces its ATPase activity [36]. These data suggest that significant and global SCS‐related changes in protein acylations are likely linked to disease presentation herein and warrant future investigation.

### 4.2 Phenotypic Differences Between the SUCLA2‐Deficient SOL and EDL Yield Investigative Opportunity for Fibre Type–Specific Mechanisms of Mitochondrial Myopathy

Most interestingly, this work revealed dramatic functional and physiological differences between muscles despite equivalent loss of SCS activity. Specifically, the more oxidative SOL muscle was more affected by A‐SCS loss than was the glycolytic EDL. Although no deficiencies were observed in the *Sucla2* ^*−/−*^ EDL ex vivo function, lowered specific twitch force and 40% reduction in maximal tetanic specific force were observed in the SUCLA2‐deficient SOL, suggesting significantly reduced muscle strength/maximum force specifically in the mutant SOL (Figure 5a). Similarly, the *Sucla2* ^−/−^ SOL exhibited a slowing of contractile kinetics, a leftward shift in the force–frequency relationship and resistance to fatigue, whereas the EDL remained largely unaffected by *Sucla2* genotype. These SOL‐specific changes are likely explained, at least in part, by the substantial shift to a slower fibre type, a phenomenon also noted in humans with mitochondrial myopathies [37].

For example, the leftward shift in the force–frequency curve may be the result of slowed rates of relaxation between muscle contractions caused by slower calcium ion kinetics [38, 39, 40]. It has been shown that the SOL has threefold to fourfold lower calcium concentrations and a different SERCA and calcium‐binding proteins within the sarcoplasmic reticulum (SR) than the EDL, which corresponds to the ‘slower’ contractile rates of these muscles [40]. With nearly double the amount of mitochondrially reliant Type 1 fibres in the SUCLA2‐deficient SOL (Figure 8), coupled with greater than threefold increase in mitochondrial content (Figure 7), increased calcium uptake in the mutant SOL may lead to reduced calcium concentrations in the SR and the even slower contractile rates. However, the decrease in maximal specific force in the *Sucla2* ^−/−^ SOL is unlikely to be explained solely by the shift towards Type 1 fibres because the differences in specific force between individual Type 1 and Type 2a fibres [39] are too low to explain the whole muscle effects observed here.

The switch to Type 1 fibre predominance and increased mitochondrial content (Figures 7, 8) may also partly explain the increased resistance to fatigue observed in the SOL. Greater mitochondrial content will increase energy supply, whereas Type 1 myosin heavy chain expressing fibres are more efficient, requiring less ATP per contraction and decreasing energy demand [41]. Although the mechanism for the fibre‐type switching in *Sucla2* mutant SOL remains to be determined and warrants further investigation, one possible mechanism may involve PPARδ signalling. The PPARδ transcription factor can drive mitochondrial production and Type 1 myosin heavy chain expression [42]. Notably, PPARδ is directly activated by free fatty acids, and lipid content is increased in the *Sucla2*‐deficient SOL, suggesting that PPARδ activity may be increased in this model (Figure 7d–e). Although further investigation is required to fully understand the mechanisms behind the divergent phenotypes observed within the SOL and EDL, these data underscore the opportunity to investigate muscle and fibre type–specific pathogenic mechanisms. Therefore, the model presented within this report provides an invaluable tool to better understand, and treat, SCS‐related mitochondrial myopathy.

## Conflicts of Interest

The authors declare no conflicts of interest.

## Supporting information


**Dataset S1** **Metabolomics**: raw values of all measured metabolites

**Dataset S2** **Behavioral Analysis**: raw output from all whole animal behavioral phenotyping assays

**Dataset S3** **Muscle Contraction Data**: raw output from ex vivo contractile studies

**Table S1** Antibody conditions for western blotting
**Table S2:** List of all antibodies and incubation conditions for fiber‐typing immunofluorescence
**Table S3:** Succinyl‐CoA synthetase activity in the mouse hindlimb muscles
**Table S4:** Western blot densitometries of SCS components in the mouse hindlimb muscles
**Table S5:** Western blot densitometries of protein acylation in the mouse hindlimb muscles
**Table S6:** Quantification of mitochondrial and lipid droplet content in electron microscopy images
**Figure S1.** Diagram of in‐house gait analysis
**Figure S2:** Equivalent levels of *Sucla2* knock‐out observed between *Sucla2* mutant genotypes
**Figure S3.** Metabolic markers and protein acylation in SUCLA2‐deficient skeletal muscle
**Figure S4:** Whole animal behavioral phenotyping
**Figure S5:** Consumption data normalized to whole body weights
**Figure S6:** Comparative analyses of fiber‐type distributions in the muscles of the hindlimb
**Figure S7:** No observed differences in whole cell concentrations of nucleotides
