# Supplementary material for: Sucla2 Knock‐Out in Skeletal Muscle Yields Mouse Model of Mitochondrial Myopathy With Muscle Type–Specific Phenotypes
Source: J Cachexia Sarcopenia Muscle. 2024 Oct 31;15(6):2729–42. doi: 10.1002/jcsm.13617 (PMC11634519; doi:10.1002/jcsm.13617)
Supplement: Supplementary file 4 — Table S1 Antibody conditions for western blotting Table S2: List of all antibodies and incubation conditions for fiber‐typing immunofluorescence Table S3: Succinyl‐CoA synthetase activity in the mouse hindlimb muscles Table S4: Western blot densitometries of SCS components in the mouse hindlimb muscles Table S5: Western blot densitometries of protein acylation in the mouse hindlimb muscles Table S6: Quantification of mitochondrial and lipid droplet content in electron microscopy images Figure S1. Diagram of in‐house gait analysis Figure S2: Equivalent levels of Sucla2 knock‐out observed between Sucla2 mutant genotypes Figure S3. Metabolic markers and protein acylation in SUCLA2‐deficient skeletal muscle Figure S4: Whole animal behavioral phenotyping Figure S5: Consumption data normalized to whole body weights Figure S6: Comparative analyses of fiber‐type distributions in the muscles of the hindlimb Figure S7: No observed differences in whole cell concentrations of nucleotides [file JCSM-15-2729-s002.docx]

**Supplemental Information**

- **DatasetS1_Metabolomics**: raw values of all measured metabolites
- **DatasetS2_BehavioralAnalysis**: raw output from all whole animal behavioral phenotyping assays
- **DatasetS3_MuscleContractionData**: raw output from *ex vivo* contractile studies
- **Supplemental Methods**
  - **Table S1:** Antibody conditions for western blotting
  - **Table S2:** List of all antibodies and incubation conditions for fiber-typing immunofluorescence
- **Table S3:** Succinyl-CoA synthetase activity in the mouse hindlimb muscles
- **Table S4:** Western blot densitometries of SCS components in the mouse hindlimb muscles
- **Table S5:** Western blot densitometries of protein acylation in the mouse hindlimb muscles
- **Table S6:** Quantification of mitochondrial and lipid droplet content in electron microscopy images
- **Figure S1.** Diagram of in-house gait analysis
- **Figure S2:** Equivalent levels of *Sucla2* knock-out observed between *Sucla2* mutant genotypes
- **Figure S3.** Metabolic markers and protein acylation in SUCLA2-deficient skeletal muscle
- **Figure S4:** Whole animal behavioral phenotyping
- **Figure S5:** Consumption data normalized to whole body weights
- **Figure S6:** Comparative analyses of fiber-type distributions in the muscles of the hindlimb
- **Figure S7:** No observed differences in whole cell concentrations of nucleotides

**Supplemental Methods**

**Genotyping**

The primer sequences used for genotyping are provided below.

*Sucla2* Forward 1: GCCTGGTTCCACTGGAGGAA

*Sucla2* Forward 2: GACGTATTATCCATTACTCAC

*Sucla2* Reverse: GCAGAAAAAGAACAGGATGC

Cre Forward: CCGGTCGATGCAACGAGTGAT

Cre Reverse: ACCAGAGTCATCCTTAGCGCC

**Western Blot Antibodies**

| **Table S1. Antibody conditions for western blotting** | | | |
| --- | --- | --- | --- |
| **Primary Antibody** | **Dilution** | **Secondary Antibody** | **Dilution** |
| Rabbit α-SUCLA2 (Gene Tex: GTX109728) | 1:20k | Goat α-Rabbit IgG | 1:30k |
| Rabbit α-SUCLG2 (GeneTex: GTX107002) | 1:10k | Goat α-Rabbit IgG | 1:30k |
| Rabbit α-SUCLG1 (GeneTex: GTX109215) | 1:10k | Goat α-Rabbit IgG | 1:30k |
| Rabbit pan α-succinyllysine (PTM Biolabs: PTM-401) | 1:1k | Goat α-Rabbit IgG | 1:30k |
| Mouse pan α-propionyllysine (PTM Biolabs: PTM-203) | 1:1k | Goat α-Mouse IgG | 1:30k |
| Mouse α-GAPDH (GeneTex: GTX627408) | 1:25k | Goat α-Mouse IgG | 1:30k |

**Table S1.** Primary and secondary antibody conditions for all western blots. Note: all secondary antibodies used were purchased from GeneTex.

**Ex Vivo Muscle Contractions**

To provide data describing the force-frequency relationships and maximal isometric force for the EDL and SOL muscles, both muscles were stimulated every 3 minutes with the following increasing pulse frequencies:

EDL: 200ms train durations at 20, 40, 60, 80, 100, 120, 140, 160, 180, and 200 Hz

SOL: 400ms train durations at 10, 20, 40, 60, 80, 100, 120, 140, 160, and 180 Hz

For the fatiguing protocols, each muscle was stimulated for 5 minutes at frequencies chosen to elicit tetanic contractions and maximal isometric force:

EDL: 180 Hz, 200ms train durations every 10 seconds

SOL: 120 Hz, 400ms train durations every 6 seconds

**Supplemental Tables and Figures**

**Immunofluorescence Staining**

**Table S2.** Primary and secondary antibodies were diluted in 0.5% BSA and 2% Normal goat serum in 1X PBS. Primary antibodies were incubated overnight at 4°C in a humidified chamber, while secondary antibodies were incubated at room temperature protected from light for one hour.

| **Table S2. List of antibodies and incubation conditions for fiber-typing immunofluorescence** | | | | | |
| --- | --- | --- | --- | --- | --- |
| **Protein** | **Primary Ab** | **Host/ Isotype** | **1° dilution** | **Secondary Ab** | **2° dilution** |
| Laminin | Sigma -  #L9393 | Rabbit polyclonal | 1:500 | Life Technologies -- #A-21244.  Alexa Fluor 647 Goat Anti-Rabbit IgG (H+L) | 1:300 |
| MyHC - slow | DSHB –  #BA-F8 | Mouse IgG2b | 1:100 | Life Technologies -- #A-21140.  Alexa Fluor 350 Goat Anti-Mouse IgG_2b_ (γ2b) | 1:300 |
| MyHC – IIa | DSHB –  #SC-71 | Mouse IgG1 | 1:100 | Life Technologies -- #A-21121.  Alexa Fluor® 488 Goat Anti-Mouse IgG_1_ (γ1) Antibody | 1:300 |
| MyHC – IIb | DSHB –  #BF-F3 | Mouse IgM | 1:100 | Life Technologies -- #A-21045.  Alexa Fluor® 546 Goat Anti-Mouse IgM (µ chain) | 1:300 |

| **Table S3. Succinyl-CoA Synthetase Activity in the Mouse Hindlimb Muscles** | | | | | | | |
| --- | --- | --- | --- | --- | --- | --- | --- |
| **Tissue** | **Experimental Results** | **ADP-Specific** | | **GDP-Specific** | | **Total Activity** | |
|  |  | **Controls** | **Mutants** | **Controls** | **Mutants** | **Controls** | **Mutants** |
| Soleus | Avg. Activity | 6.672 | 1.540 | 2.221 | 3.256 | 8.893 | 4.796 |
|  | *St. Dev.* | *0.732* | *1.162* | *0.819* | *0.487* | *1.351* | *1.512* |
| EDL | Avg. Activity | 6.253 | 2.218 | 2.448 | 3.477 | 8.701 | 4.827 |
|  | *St. Dev.* | *0.919* | *1.629* | *1.833* | *1.140* | *1.793* | *1.674* |
|  | **Average FC**  **p-value** | **0.293**  **0.002** | | **1.443**  **0.664** | | **0.547**  **0.004** | |

**Table S3** provides the kinetic experimental results for the nucleotide-specific enzymatic activities in the soleus and EDL of *Sucla2* control and mutant mice. Average activities shown for each tissue are expressed in nmol/min/mg protein, with standard deviations (St.Dev.) provided. The average fold-change (FC, mutant/control) is a combined average of the genotype-specific changes observed in both tissues, with a pooled p-value calculated via standard unpaired t-tests. Controls = Sucla2^+/+^, *HSA*-Cre positive and Mutants = Sucla2^Flox/Flox^, *HSA*-Cre positive. N = 3-4 per genotype.

| **Table S4. Western Blot Densitometries of SCS Components in the Mouse Hindlimb Muscles** | | | | | | | |
| --- | --- | --- | --- | --- | --- | --- | --- |
| **Tissue** | **Experimental Results** | **SUCLA2** | | **SUCLG2** | | **SUCLG1** | |
|  |  | **Controls** | **Mutants** | **Controls** | **Mutants** | **Controls** | **Mutants** |
| Soleus | Average | 2.369 | 0.073 | 0.266 | 1.793 | 1.907 | 0.365 |
|  | *St. Dev.* | *0.663* | *0.028* | *0.092* | *0.914* | *0.155* | *0.184* |
| EDL | Average | 1.491 | 0.075 | 0.266 | 2.565 | 1.242 | 0.473 |
|  | *St. Dev.* | *0.356* | *0.039* | *0.250* | *1.261* | *0.640* | *0.113* |
|  | **Average FC**  **p-value** | **0.040**  **0.00004** | | **8.194**  **0.005** | | **0.286**  **0.015** | |

**Table S4** depicts the nonnormalized western blot densitometry results for both the soleus and EDL of *Sucla2* mutant and control mice. Average band intensity for each SCS component (divided by the band intensity of the GAPDH loading control) are provided for each tissue, as well as the standard deviation. An overall average of the genotype-specific (mutant/control) fold-change in both tissues is also provided, with a pooled p-value (calculated by standard unpaired t-tests) showing significant changes for the expression of all SCS components in both tissues. Controls = Sucla2^+/+^, HSA-Cre positive, and Mutants = either Sucla2^Fl/∆^ or Sucla2^Fl/Fl^ and HSA-Cre positive. N = 5 per genotype.

| **Table S5. Western Blot Densitometries of Protein Acylation in the Mouse Hindlimb Muscles** | | | | | | |
| --- | --- | --- | --- | --- | --- | --- |
| **Tissue** | **Experimental Results** | **Succinyllysine** | | **Propionyllysine** | | |
|  |  | **Controls** | **Mutants** | **Controls** | | **Mutants** |
| Soleus | Average | 0.158 | 2.554 | 0.533 | | 1.508 |
|  | *St. Dev.* | *0.040* | *2.568* | *0.190* | | *0.700* |
| EDL | Average | 0.074 | 2.233 | 0.487 | | 2.134 |
|  | *St. Dev.* | *0.025* | *1.690* | *0.211* | | *1.212* |
|  | **Average FC**  **p-value** | **23.170**  **0.046** | | | **3.606**  **0.009** | |

**Table S5** outlines the nonnormalized western blot densitometry results for protein acylation in both the soleus and EDL of *Sucla2* mutant and control mice. Average band intensity was calculated from the highest intensity band via ImageJ, and normalized to the GAPDH loading control. An overall average of the genotype-specific (mutant/control) fold-change in both tissues is also provided, with a pooled p-value (calculated by standard unpaired t-tests) showing significant changes in both protein succinylation and propionylation in skeletal muscle (although the 16-fold increase in lysine succinylation in the soleus was only trending with a p-value of 0.07). Controls = Sucla2^+/+^, HSA-Cre positive, and Mutants = either Sucla2^Fl/∆^ or Sucla2^Fl/Fl^ and HSA-Cre positive. N = 5 per genotype.

| **Table S6. Quantification of Mitochondrial and Lipid Droplet Content in TEM Images** | | | | | | |
| --- | --- | --- | --- | --- | --- | --- |
| **Image ID** | **Soleus** | | | **EDL** | | |
|  | **Lipid Area** | **Mito Area** | **Field Area** | **Lipid Area** | **Mito Area** | **Field Area** |
| **Control1_1** | 0 | 34.891 | 280.018 | 0 | 23.488 | 275.292 |
| **Control1_2** | 0 | 33.603 | 275.607 | 1.815 | 22.656 | 221.287 |
| **Control1_3** | 0 | 32.28 | 235.372 | 0.574 | 32.573 | 275.922 |
| **Control1_4** | 0.074 | 32.066 | 280.659 | 2.252 | 18.655 | 264.263 |
| **Control2_1** | 0.387 | 35.358 | 279.344 | 0.213 | 18.594 | 275.049 |
| **Control2_2** | 0.17 | 27.797 | 236.314 | 0 | 17.852 | 275.116 |
| **Control2_3** | 0.202 | 37.913 | 275.363 | 0.111 | 42.014 | 270.685 |
| **Control2_4** | 0.624 | 44.181 | 274.873 | 0.043 | 58.738 | 274.134 |
| **Control3_1** | 1.804 | 30.978 | 263.662 | 0 | 16.849 | 195.932 |
| **Control3_2** | 0.336 | 22.782 | 257.912 | 0.237 | 12.792 | 280.659 |
| **Control3_3** | 1.053 | 46.603 | 232.657 | 0.12 | 20.044 | 234.037 |
| **Control3_4** | 0.74 | 35.568 | 275.049 | 0.311 | 11.196 | 270.685 |
| **Control4_1** | 0.356 | 22.634 | 266.497 | 0.422 | 29.93 | 266.497 |
| **Control4_2** | 0.797 | 43.727 | 275.607 | 1.115 | 26.338 | 271.824 |
| **Control4_3** | 1.246 | 36.141 | 270.48 | 0.986 | 11.881 | 270.685 |
| **Control4_4** | 1.107 | 29.506 | 280.018 | 0.169 | 29.749 | 274.873 |
| ***AVERAGE*** | ***0.556*** | ***34.127*** | ***266.214*** | ***0.523*** | ***24.584*** | ***262.309*** |
| ***ST.DEV*** | ***0.531*** | ***6.887*** | ***16.813*** | ***0.682*** | ***12.312*** | ***23.840*** |
| **Mutant1_1** | 3.566 | 162.162 | 266.261 | 2.343 | 63.342 | 280.339 |
| **Mutant1_2** | 6.31 | 29.854 | 266.192 | 1.53 | 9.556 | 270.272 |
| **Mutant1_3** | 5.676 | 85.79 | 274.63 | 0.135 | 63.231 | 245.827 |
| **Mutant1_4** | 0.656 | 130.164 | 275.922 | 0.127 | 38.814 | 197.581 |
| **Mutant2_1** | 3.889 | 79.326 | 271.304 | 1.929 | 24.406 | 265.939 |
| **Mutant2_2** | 1.647 | 117.286 | 275.363 | 0.603 | 31.45 | 274.243 |
| **Mutant2_3** | 2.302 | 111.335 | 270.755 | 0.41 | 60.386 | 260.065 |
| **Mutant2_4** | 1.503 | 103.559 | 270.033 | 0.91 | 35.034 | 266.801 |
| **Mutant3_1** | 1.303 | 104.286 | 280.091 | 0.972 | 80.76 | 275.607 |
| **Mutant3_2** | 0.648 | 76.436 | 274.873 | 3.711 | 16.649 | 280.018 |
| **Mutant3_3** | 1.454 | 100.589 | 280.659 | 0.873 | 30.382 | 274.63 |
| **Mutant3_4** | 1.238 | 136.002 | 270.376 | 0.335 | 23.372 | 222.554 |
| **Mutant4_1** | 1.643 | 103.361 | 190.019 | 0.147 | 32.238 | 216.925 |
| **Mutant4_2** | 1.136 | 121.915 | 265.552 | 0.193 | 55.381 | 268.096 |
| **Mutant4_3** | 1.734 | 109.832 | 274.873 | 2.424 | 42.387 | 270.685 |
| **Mutant4_4** | 0.907 | 133.812 | 243.428 | 0.175 | 13.882 | 267.106 |
| ***AVERAGE*** | ***2.226*** | ***106.607*** | ***265.646*** | ***1.051*** | ***32.829*** | ***258.543*** |
| ***ST.DEV*** | ***1.730*** | ***30.185*** | ***21.907*** | ***1.059*** | ***20.506*** | ***24.783*** |

**Table S6** provides the raw quantitation of lipid droplet area, mitochondrial area, and total field area in µm^2^ in images taken via transmission electron microscopy (TEM). Four representative images were taken of the soleus and EDL for each animal (n=4 per genotype) at 6800x in different areas of the section grid. Structures from each image (n=16 per genotype) were then measured and cumulative area was quantified via ImageJ Software. Controls = Sucla2^+/+^, *HSA*-Cre positive and Mutants = Sucla2^Fl/Fl^, *HSA*-Cre positive.


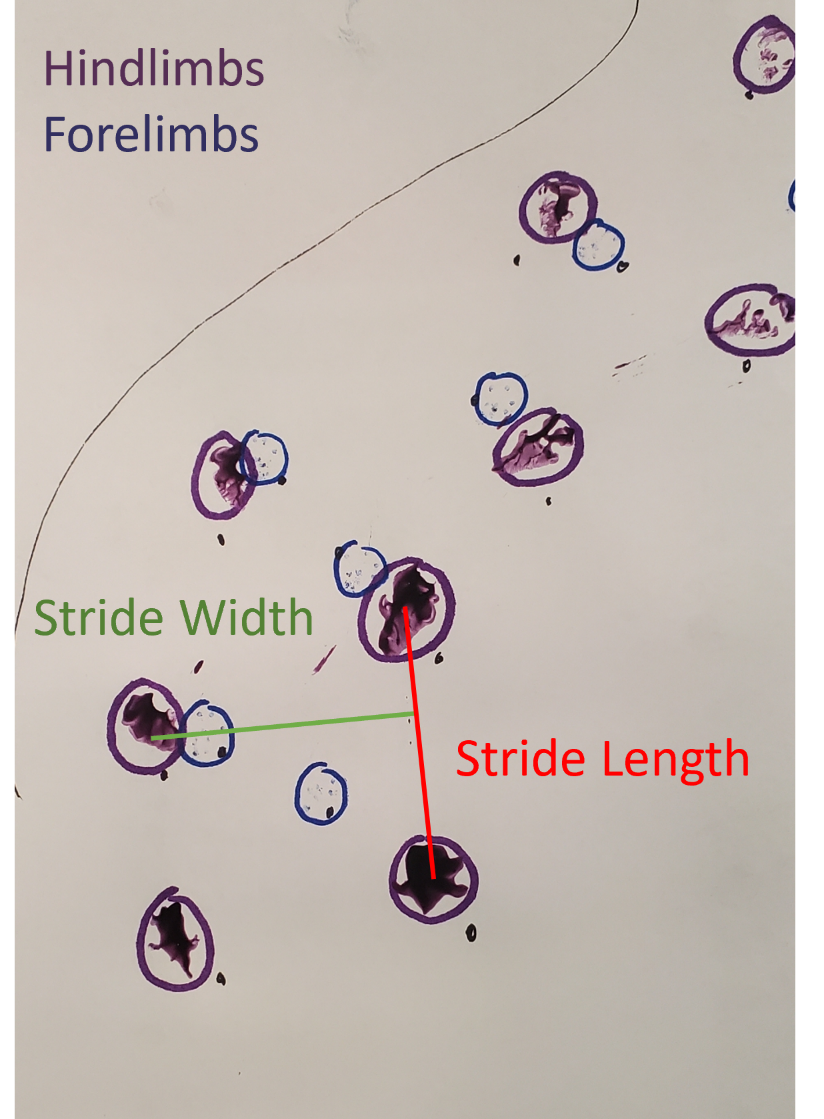


**Figure S1. Diagram of in-house gait analysis**. Hindlimbs footpads were pressed into animal-safe purple paint, and forelimbs footpads were pressed into an animal-safe blue inkpad. Mice were placed on a plain white sheet of paper and allowed to take at least four consecutive steps. Stride length (green) was determined by the distance between the center of the footpad of the same foot from one step to the next consecutive step. Stride width (red) was measured as the distance perpendicular to the center of the stride length of one side, to the center of the footpad of the opposite foot. Measurements of all steps were quantified using ImageJ.


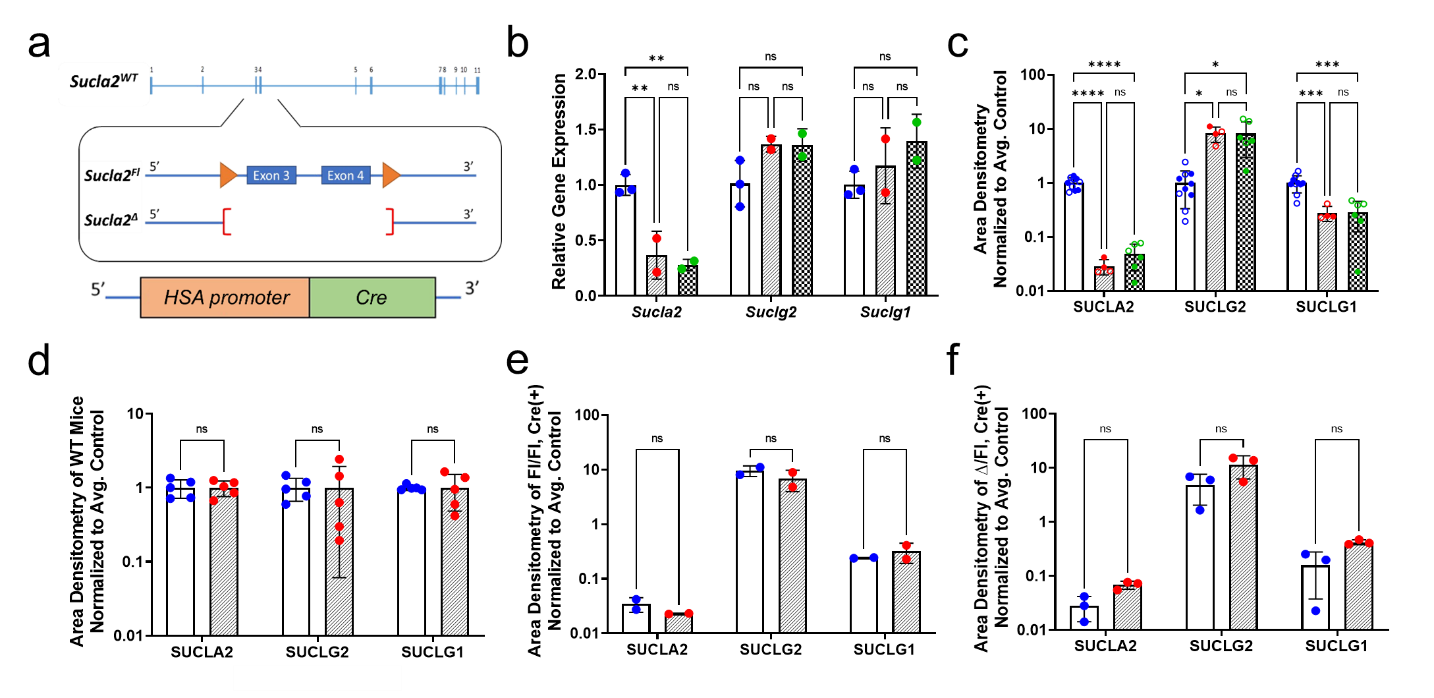


**Figure S2. Equivalent levels of *Sucla2* knock-out are observed between *Sucla2* mutant genotypes and between muscles. (a)** Representations of the *Sucla2* alleles used within this report. CRISPR-Cas9 technology was used to generate the *Sucla2* Floxed alleles (*Sucla2^Fl^*) with loxP sites flanking exons three and four of the endogenous *Sucla2* gene. As a byproduct of the CRISPR event and non-homologous end-joining DNA repair, a constitutive deletion allele (*Sucla2^∆^*) was recovered. Mutant *Sucla2* genotypes used herein are either homozygous for the floxed allele or compound heterozygous for the floxed allele and the deletion. All animals within this study are positive for the *HSA*-Cre transgene. **(b)** RT-qPCR and **(c)** western blot analysis of mouse hindlimb muscles revealed no differences in relative gene or protein expression between the mutant genotypes. In the bar graphs of the top panel (b-c), *Sucla2^+/+^, HSA*-Cre(+) data are in blue (clear bars), *Sucla2^Fl/Fl^, HSA-Cre(+)* data are red (slanted pattern), and *Sucla2^∆/Fl^, HSA-Cre(+)* data are green (checkered pattern). RT-qPCR data was collected from whole hindlimb skeletal muscle in p0 mouse pups, and western blot analysis was performed on soleus (filled circles) and EDL (open circles). **(d-f)** Western blot analysis also showed no significant differences in the relative levels of protein expression of SCS components between the soleus (blue) and EDL (red) in any *Sucla2* genotype: **(d)** *Sucla2^+/+^, HSA-*Cre(+) **(e)** *Sucla2^Fl/Fl^, HSA-*Cre(+) **(f)** *Sucla2^∆/Fl^, HSA*-Cre(+). All data are presented as means ± SD, and significant differences are depicted by asterisks, where * = p<0.05, ** = p<0.02, *** = p<0.001, and ****p<0.0001 by 2-way ANOVA followed by Tukey post-hoc multiple comparisons analysis.


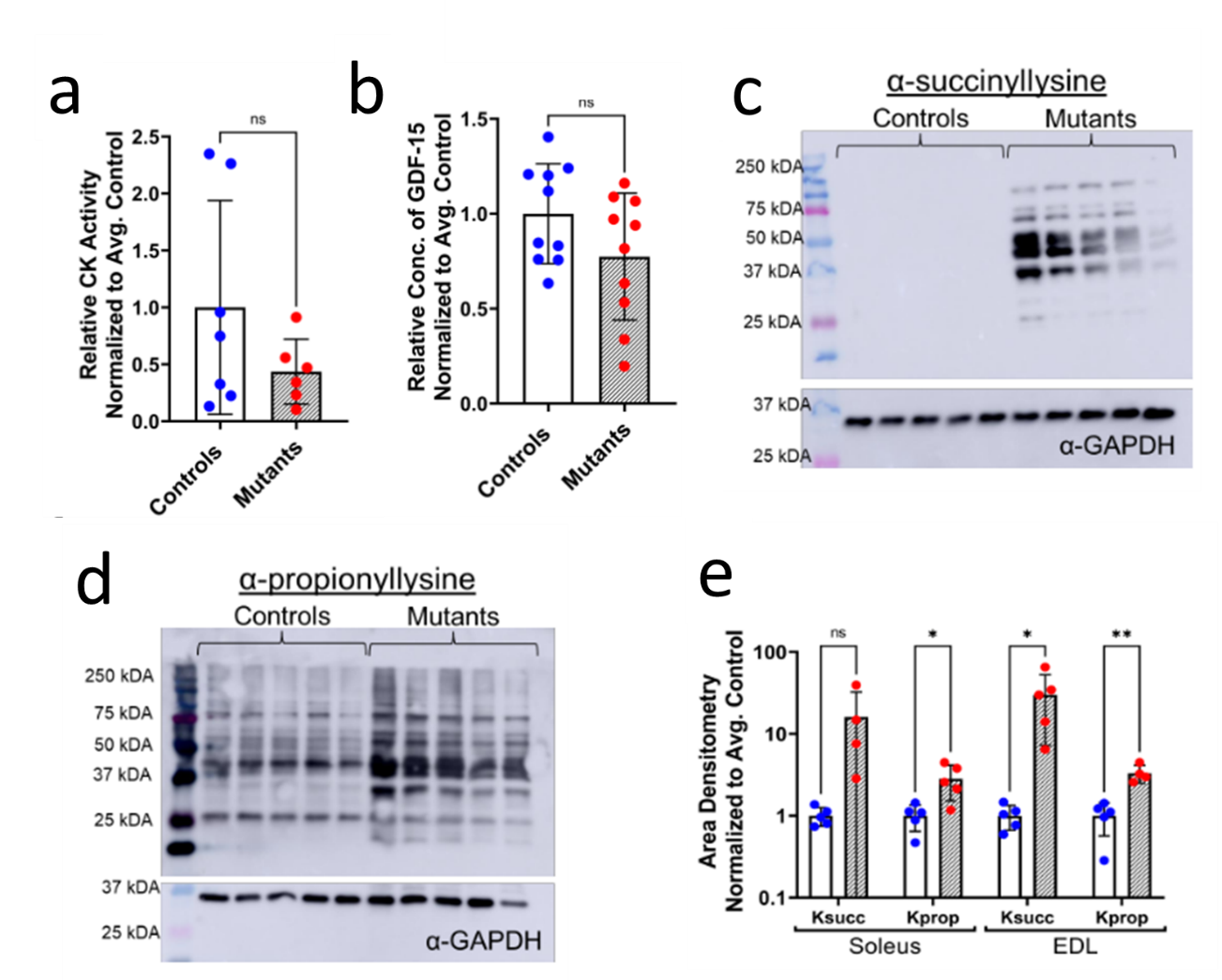


**Figure S3. Metabolic markers and protein acylation in SUCLA2-deficient skeletal muscle.** **(a)** Enzymatic activity levels of creatine kinase measured according to manufacturer’s protocols (Abcam: ab155901), n=7. **(b)** Serum concentrations of GDF-15 (Abcam: ab216947) measured via ELISA. **(c-d)** Western blot analysis against succinyllysine **(c)** and propionyllysine **(d)** protein modifications in the SOL. **(e)** Densitometry quantification calculated using ImageJ software of western blots for both modifications in the SOL and EDL, with nonnormalized results provided in Table S5. The Control genotype is *Sucla2^+/+^, HSA*-Cre positive, and Mutant mice are either *Sucla2^Fl/Fl^* or *Sucla2^Δ1/Fl^* and *HSA*-Cre positive. Significant differences are depicted by asterisks, where * = p<0.05, ** = p<0.01, *** = p<0.001, and **** = p<0.0001 by multiple unpaired t-tests, with p-values corrected for multiple testing via false discovery rate (FDR).


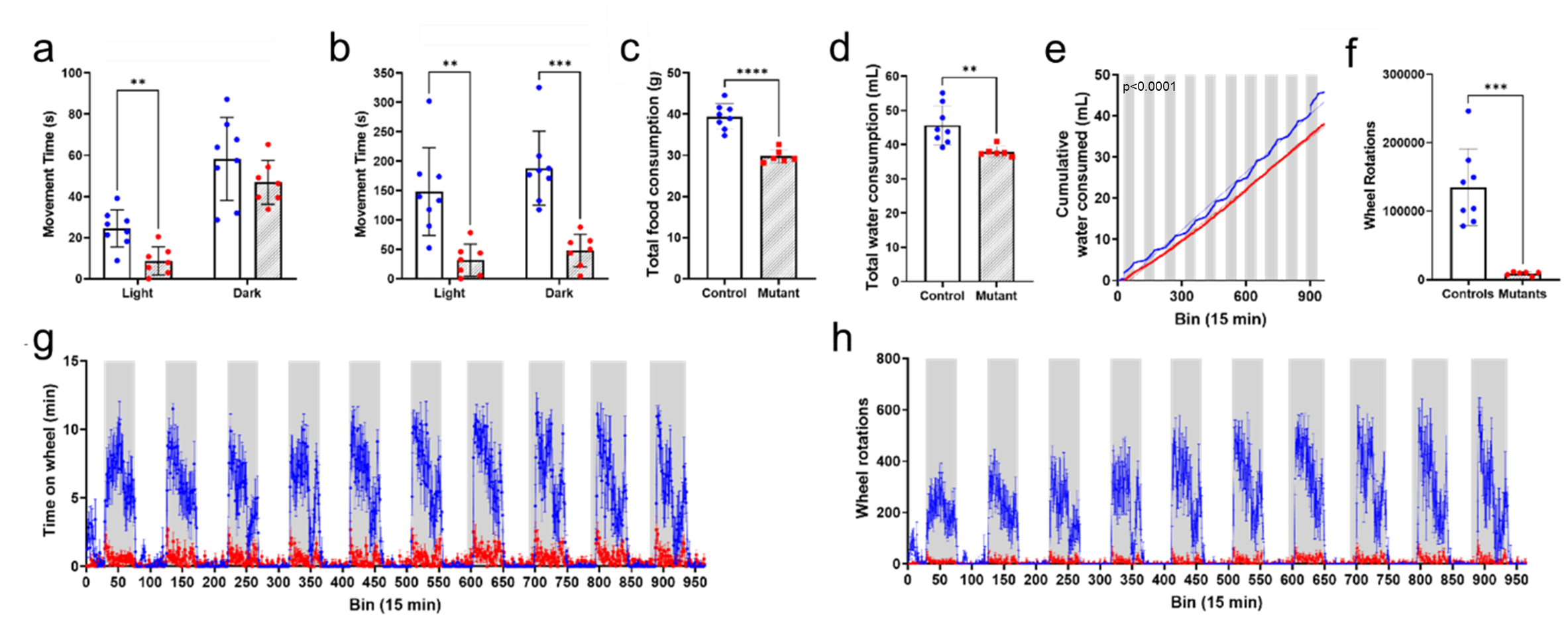


**Figure S4. Whole animal behavioral phenotyping.** Figures **a-b** depict data collected from an open field analysis, where the mice had access to both a light and dark environment. **(a)** Total stereotypy movement time. **(b)** Total vertical movement time. Figures **c-e** show food and water consumption data gathered from telemetry housing. Bar graphs show **(c)** total food and **(d)** water consumed after 10 days in telemetry housing, and **(e)** x-y plots show cumulative consumption of water over time. Figures **f-h** illustrate wheel running activity data gathered from telemetry housing. **(f)** Bar graph shows the average total wheel rotations. Longitudinal running wheel behavior, including **(g)** time spent on the wheel in 15-minute bins and **(h)** wheel rotations in 15-minute bins are also provided. Grey and white columns indicate 12-hour periods of light and dark, respectively. For all data shown, Controls (blue) = Sucla2^+/+^, *HSA*-Cre positive, and Mutants (red) = either Sucla2^Fl/∆^ or Sucla2^Fl/Fl^ and *HSA*-Cre positive. N = 7-8 per genotype. All data presented are represented as means ± SD, and statistically significant differences are depicted by asterisks, where * = p<0.05, ** = p<0.02, *** = p<0.001, and ****p<0.0001 by either standard unpaired t-tests or Tukey-post hoc test after 2-way ANOVA. Statistical comparison of consumption behavior was conducted via linear regression analysis, with the p-value representing significantly differing slopes.


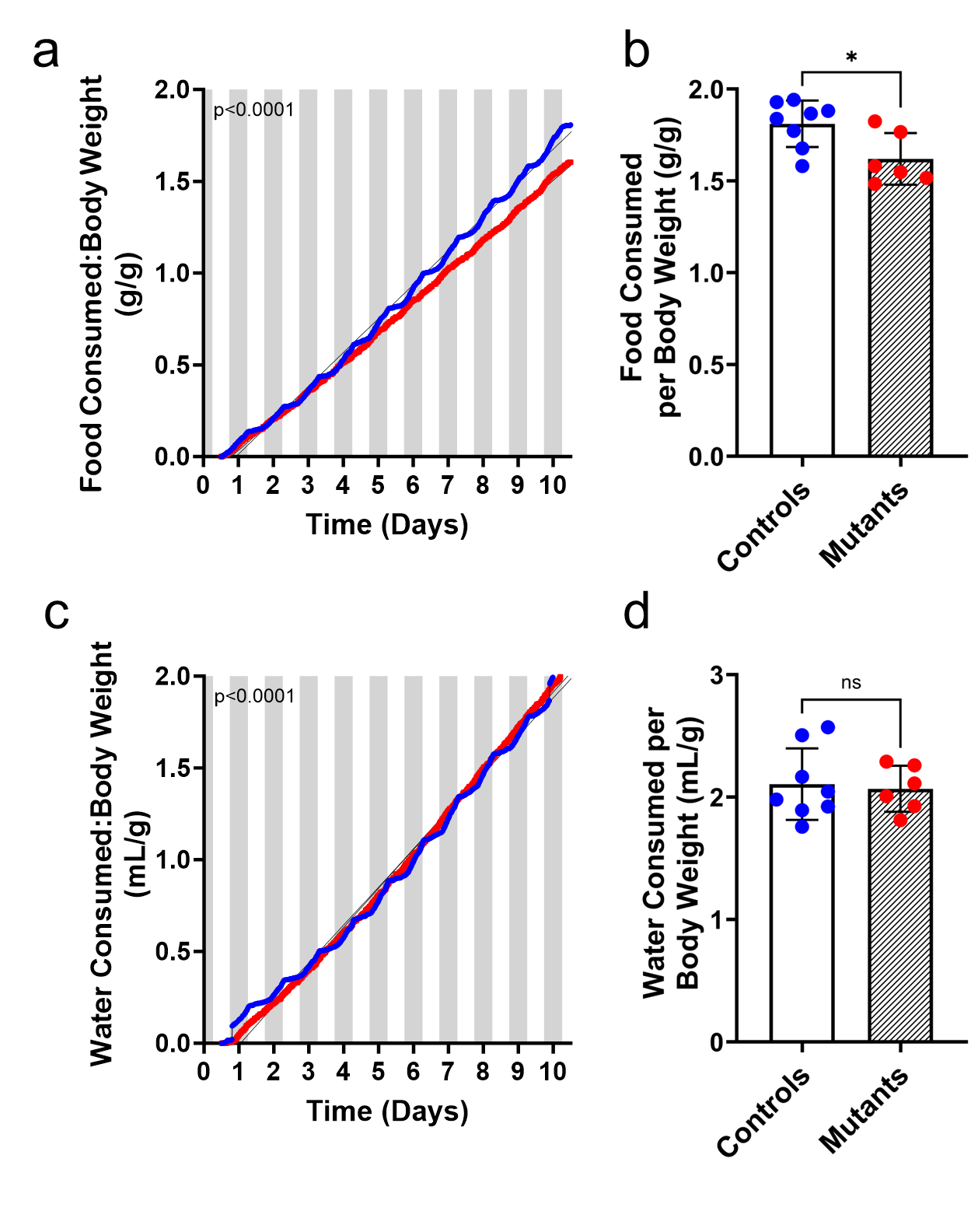


**Figure S5. Consumption data normalized to whole body weights.** Figures **(a)** and **(c)** depict the longitudinal consumption data, collected every 15 minutes, normalized to single body weight measurements taken at the beginning of the data collection (Day 1). P-values represent genotype-specific differences in slopes calculated via linear regression analysis. Cumulative amounts of **(b)** food and **(d)** water consumed were also normalized to the single body weight measurement. Grey and white columns indicate 12-hour periods of light and dark, respectively. For all data shown, Controls (blue) = Sucla2^+/+^, *HSA*-Cre positive, and Mutants (red) = either Sucla2^Fl/∆^ or Sucla2^Fl/Fl^ and *HSA*-Cre positive. N = 7-8 per genotype. Data presented in bar graphs are represented as means ± SD, and significant differences are depicted by asterisks, where * = p<0.05 by unpaired t-test.

**
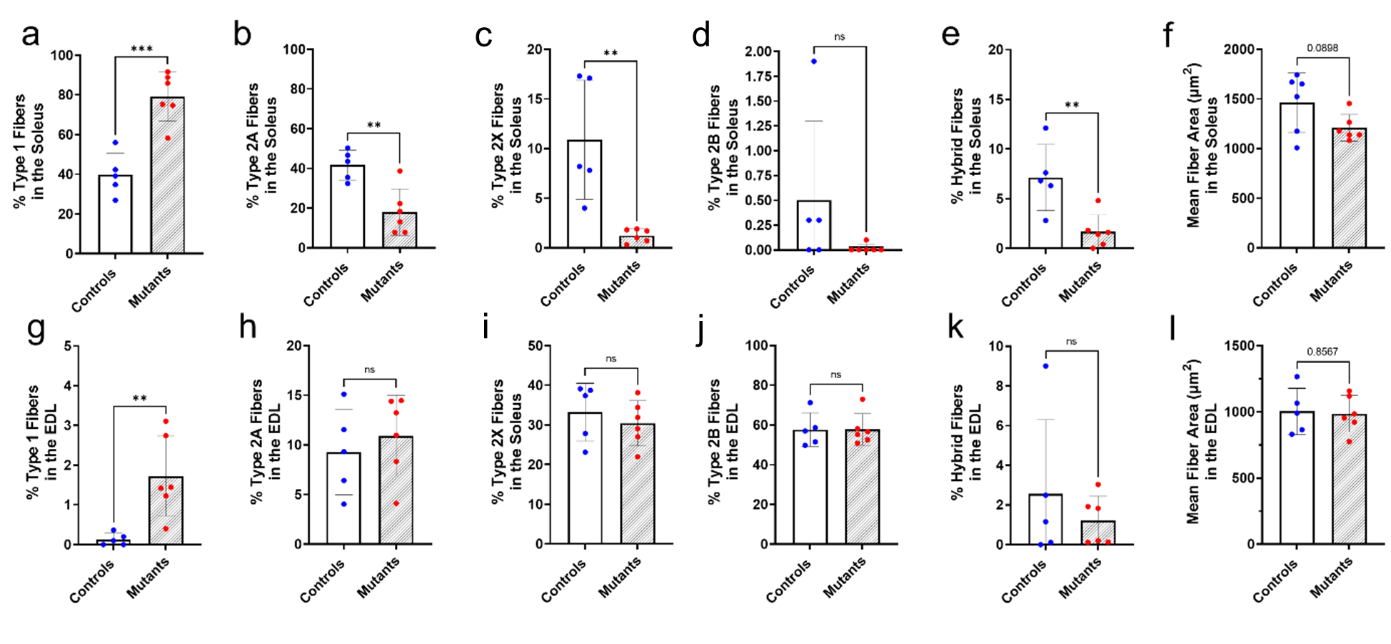
Figure S6. Comparative analyses of fiber-type distributions in the muscles of the hindlimb.** The top row of figures outlines the percent contribution of **(a)** type 1, **(b)** type 2A, **(c)** type 2X, **(d)** type 2B, and **(e)** hybrid-staining fibers in the soleus, as well as **(f)** the average fiber size (µm^2^) of all fiber types within the soleus of *Sucla2* mutant and control mice. The bottom row of figures outlines the percent contribution of **(g)** type 1, **(h)** type 2A, **(i)** type 2X, **(j)** type 2, and **(k)** hybrid-staining fibers in the EDL, as well as **(l)** the average fiber size (µm^2^) of all fiber types within the EDL of *Sucla2* mutant and control mice. All data are presented as means ± SD, and significant differences are depicted by asterisks, where ** = p<0.02 and *** = p<0.001 by standard unpaired t-tests. Controls (blue) = Sucla2^+/+^, *HSA*-Cre positive, and Mutants (red) = Sucla2^Fl/Fl^ , *HSA*-Cre positive. N = 5-6 per genotype.


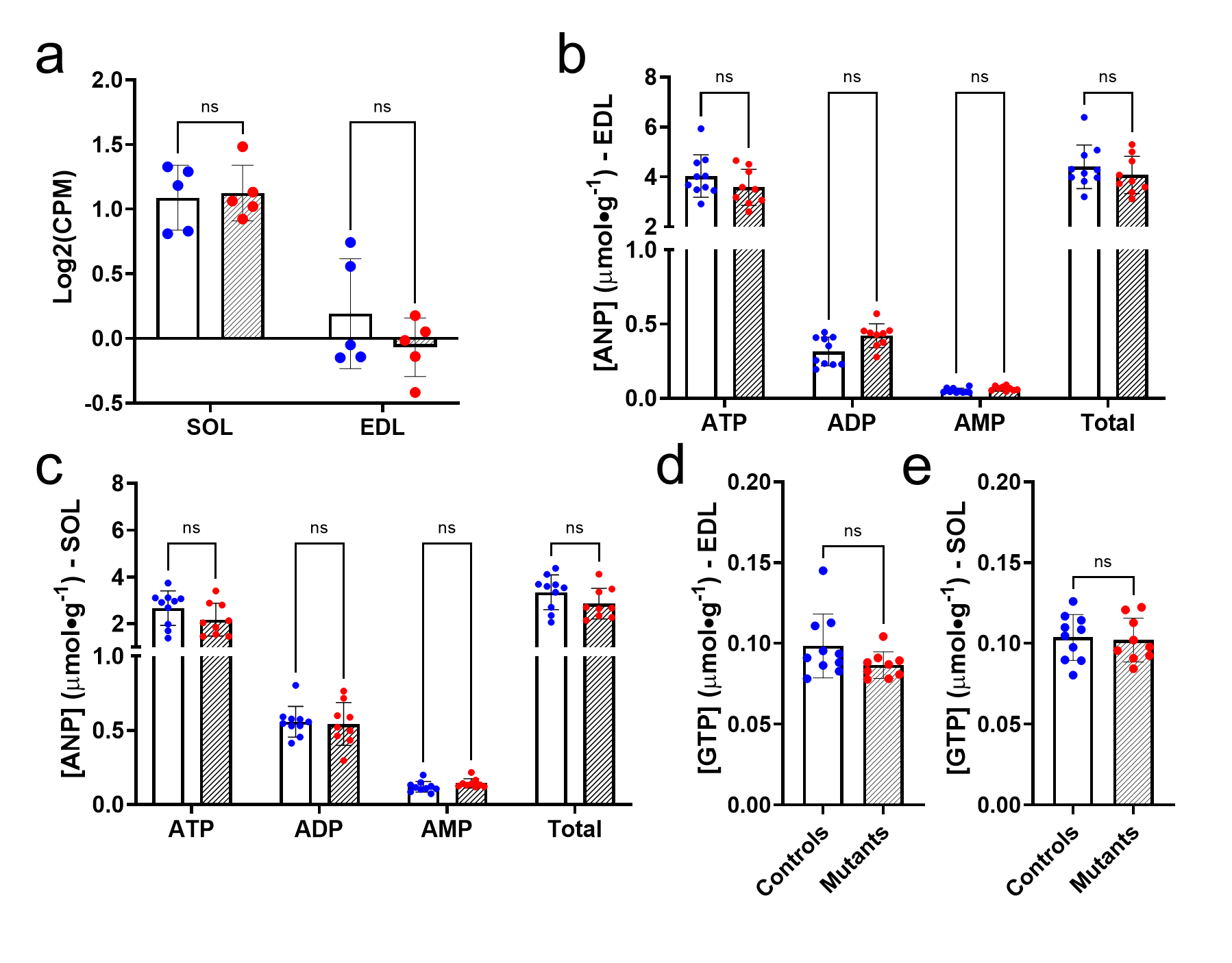


**Figure S7. Mice deficient for SUCLA2 do not exhibit differences in whole cell concentrations of nucleotides. (a)** Differential expression analysis of *Nme4* transcript expression measured via bulk RNA-sequencing of total RNA isolated from SOL and EDL muscles of *Sucla2* WT (blue) and KO (red) mice. Pooled libraries were sequenced with 2x150bp paired-end configuration on an Illumina NovaSeq 6000 sequencer. **(b-c)** Whole cell concentrations of adenine nucleotides (ANP, where “N” represents either mono-, di-, or triphosphates) measured in the **(b)** EDL and **(c)** SOL. **(d-e)** Whole cell concentrations of guanine triphosphate (GTP) measured in the **(d)** EDL and **(e)** SOL. All nucleotides measurements were calculated using an ultra-performance liquid chromatography (UPLC) method as described [#]. All data are presented as means ± SD. No significant differences were observed following two-way ANOVA with Sidak multiple comparisons post-hoc test to assess genotype effect (a-c). No significant differences were observed in guanine nucleotides following standard unpaired t-tests (d-e). Controls (blue) = Sucla2^+/+^, *HSA*-Cre positive, and Mutants (red) = Sucla2^Fl/Fl^ , *HSA*-Cre positive. N = 9-10 per genotype.
